# Carvacrol ameliorates cyclophosphamide-induced rat premature ovarian failure and uterine fibrosis via regulating PI3K/AKT/FOXO3a signaling pathway

**DOI:** 10.1186/s13048-025-01880-3

**Published:** 2025-12-09

**Authors:** Zeinab A. El-Gendy, Seham Samir Soliman, Mohamed S. Aly, Sara M. Baraka

**Affiliations:** 1 Department of Pharmacology, Medical Research and Clinical Studies Institute, National Research Centre, Dokki, Giza, 12622 Egypt; 2 Department of Animal Reproduction and Artificial Insemination, Veterinary Research Institute, National Research Centre, Dokki, Cairo, 12622 Egypt; 3 Chemistry of Natural Compounds Department, National Research Centre, Giza, 12622 Egypt

**Keywords:** Cyclophosphamide, Ovarian toxicity, Carvacrol, FOXO3a, Oxidative stress

## Abstract

**Background:**

Premature ovarian failure (POF) and ovarian reserve loss are common problems associated with cancer therapies. Carvacrol (Carva) showed significant efficacies in ameliorating several toxicological impacts and complications associated with chemotherapeutic agents. Thus, this study was established to speculate on the efficacy of Carva against cyclophosphamide (Cyclo)-provoked POF.

**Methods:**

A twenty-four mature female rats were randomly and equally allocated into four groups; normal group, Carva (15 mg/kg/day/orally/3 weeks) group, Cyclo (75 mg/kg/i.p/once weekly/3 weeks) group, and Carva + Cyclo group. Assessments of serum sex hormones, as well as ovarian oxidative stress-related pathways, along with histopathological alterations in ovary and uterine tissues, have been investigated. In addition, monitoring the rats’ estrous cycle was performed via vaginal cytology.

**Results:**

Our findings revealed that Carva ameliorated the disturbance of rats’ estrous cycle, as well as loss of rats’ body weight, and reproductive tract weight brought on by Cyclo. In addition, Carva administration modulated the disturbances in serum Anti-Mullerian hormone, estradiol, total estrogen, progesterone, follicle-stimulating hormone, and luteinizing hormone that resulted from Cyclo toxicity. The incidence of oxidative stress following Cyclo was significantly corrected by Carva, as indicated by a decline in ovarian malondialdehyde content and an increase in glutathione level. Carva effectively abrogated the increase in phosphorylated forms of protein kinase B (AKT), forkhead box protein O3a (FOXO3a), phosphoinositide 3-kinases (PI3K), and phosphatase and tensin homolog protein content in the ovarian tissue following Cyclo. Moreover, Carva ameliorated the structural changes of Cyclo in the ovarian and uterus tissues, and collagen condensation in the uterus. A marked preservation in the follicle count was recorded in the Cyclo + Carva group. The immunohistochemical findings revealed the hyper-expression of p-AKT along with activation of caspase 3 in the ovarian regions of Cyclo group, which is down-regulated by Carva.

**Conclusion:**

Hence, it could be concluded that Carva could preserve rat ovarian function from the degenerative impacts of Cyclo treatment by lessening the oxidative and apoptotic insult via regulating the PI3K/AKT/FOXO3a pathways in the ovarian tissues, along with successful attenuation of uterine fibrosis.

**Supplementary Information:**

The online version contains supplementary material available at 10.1186/s13048-025-01880-3.

## Introduction

Premature ovarian failure (POF) and ovarian reserve loss are common problems associated with cancer therapies [1]. It is frequently brought on by chemical, autoimmune, or genetic abnormalities [2]. Numerous investigations have demonstrated that chemotherapeutic drugs can cause reproductive dysfunction through apoptosis, ovarian shrinkage, follicle destruction, and altered angiogenesis [3].

Cyclophosphamide (Cyclo), a chemotherapeutic alkylating anti-tumor drug that is frequently used in clinical settings, is one of the most toxic agents to gonads. It is also used in treating rheumatic immune diseases as an immunosuppressant [4, 5]. The main pharmacological effect of Cyclo, which belongs to a group of medications that do not target specific cell cycles, is the cross-linking of DNA in tumor cells, which prevents DNA synthesis, eradicates tumor ones, and also damages multiplying cells at every stage of the cell cycle [6]. Consequently, this drug adversely affects living, active germ cells. Prior research has demonstrated that Cyclo may have negative consequences linked to the decrease in follicle storage in female tumor patients of reproductive age [7].

One of the primary causes brought on by Cyclo is oxidative stress, which lowers glutathione levels in the ovary and causes granulosa cell apoptosis, both of which can damage follicles [8]. Additionally, it is theorized that Cyclo can over-activate the phosphoinositide 3-kinases/phosphatase and tensin homolog/protein kinase B (PI3K/ PTEN/Akt) pathway to promote oocyte death. This can then result in ovarian shrinkage, an increase in atretic follicles, early follicular pool depletion, and ovarian interstitial fibrosis [9]. In vitro study using human ovarian xenograft models has demonstrated that Cyclo triggers the apoptotic cascade by causing DNA breaks in primordial follicles [10]. Moreover, prior studies reported uterine damage following Cyclo administration in rats [11–13].

Carvacrol (Carva), is a monoterpene molecule that is present in the essential oils of aromatic plants with a distinctive oregano scent, like pepperwort, thyme, and wild bergamot [14]. The Food and Drug Administration authorized its usage as a preservative in the food industry due to its low dosage safety profile. It has a lot of potential for creating innovative treatment strategies to treat illnesses in people [15]. Numerous investigations have shown that Carva exhibits potent pharmacological and biological effects, including hepatoprotective, antifungal, anticancer, antibacterial, anti-inflammatory, antioxidant, spasmolytic, and vaso-relaxant, both in vivo and in vitro [14]. Several studies demonstrated Carva’s ameliorative benefits, which included ischemia/reperfusion [16], and gentamicin-induced nephrotoxicity [17]. It also inhibits liposome phospholipid peroxidation and has a higher antioxidant activity than various synthetic antioxidants [18].

It is worth mentioning that Carva showed significant efficacies in ameliorating several toxicological impacts and complications associated with chemotherapeutic agents. For instance, Bozkrut et al. endorsed the protective effect of Carva against renal damage brought on by methotrexate (a type of chemotherapeutic agent) [19]. Additionally, prior studies validated the beneficial impacts of Carva in attenuating the kidney, heart, and testes dysfunctions accompanying Cyclo administration in rats [20–22] through diminishing oxidative insult. It has been noted that the modulation of the PI3K/AKT pathway was informed to be a significant effector of Carva’s action to antagonize the renal toxicity induced by cisplatin (a well-known chemotherapeutic drug) [23] Furthermore, Liu et al. documented that Carva can ameliorate diabetic cardiomyopathy in experimental rats through regulating the PI3K/AKT pathway [24].

Moreover, a former investigation reported that Carva could promote cell cycle arrest in human breast cancer in vitro model by modulating the PI3K/AKT pathway [25]. Therefore, we hypothesized that regulation of the PI3K/AKT pathway could be a chief contributor to Carva’s potential against ovarian failure. Hence, this research intends to elucidate the possible protective influence of Carva against Cyclo-induced ovarian damage in rats. Notably, the underlying molecular mechanisms of Carva were conjectured, particularly those about oxidative stress-initiated PI3K/AKT/forkhead box protein O3a (FOXO3a) signaling pathways, and apoptosis in the rat ovarian tissues. Further, our research aimed to evaluate whether Carva treatment could maintain primordial follicle reserve, and hormonal balance, prevent oxidative damage and apoptosis in rat ovaries exposed to cyclophosphamide, and speculate on whether phosphorylated PI3K, AKT, and FOXO3a mediate Carva’s actions. Lastly, the histopathological alterations in the ovarian and uterus tissues following Cyclo exposure and Carva treatment have been evaluated.

## Materials and methods

### Drugs and chemicals

Cyclo (1 g, Endoxan, Bater Oncology, Germany) used for induction of POF, was dissolved in saline and intraperitoneally injected at a dose of 75 mg/kg rat bw once a week/3 weeks (Safwat et al., 2022). Carva utilized in this study was obtained from Sigma Aldrich, St. Louis MO, USA. The other chemicals consumed in this study were in a high analytical grade.

### Animals

A total of twenty-four mature female albino Wistar rats (12 weeks old) weighing 130–150 g were employed in this investigation. The animals were procured from the National Research Centre’s animal house (NRC, Giza, Egypt), and housed in polypropylene cages under standard environmental circumstances with a suitable temperature of 21 ± 3℃ and humidity ranges of 60%-70% with 12 h cycle of light and dark. Free access to food and water was allowed for the experimental animals during the study.

The sample size was calculated using G-Power software version 3.1.9.4 (Fraz faul, Germany). The primary result was predetermined to be a decrease in the amount of Anti-Müllerian Hormone (AMH), which is a blood test measure of ovarian reserve, or the quantity of viable eggs that remain. AMH levels that are noticeably low or undetectable may indicate ovarian failure, sometimes referred to as primary ovarian insufficiency. In addition to gradually declining with age, ovarian-damaging treatments like chemotherapy or radiation can also lower AMH levels, which may result in POF. Prior data indicated a difference in serum AMH in the Cyclo-induced POF group, compared to the normal group [26]. Six animals were assigned to each study group to achieve an effect size of 0.8764 with a continuity correction and a study power of 95% (1 – β error probe) with a two-sided α = 0.05.

#### Ethics

The animal experimental studies were approved by the Medical Research Ethics Committee (MERC), National Research Centre, Giza, Egypt (Ethical Approval NO. 3444052023). The committee approved the animal studies, verifying their alignment with the instructions outlined in the ARRIVE guidelines and the Guide for Care and Use of Laboratory Animals (8th ed. 2011).

### Experimental protocol

The study was designed as a controlled, randomized experimental study. Animals were randomly assigned to experimental groups using a computer-generated random sequence. Allocation concealment was maintained by having a researcher not involved in interventions or outcome assessments perform the group assignments. Outcome assessments were performed in a blinded manner: histological scoring, western blot densitometry, and ELISA measurements were carried out by investigators unaware of group allocation.

**Fig. 1:**
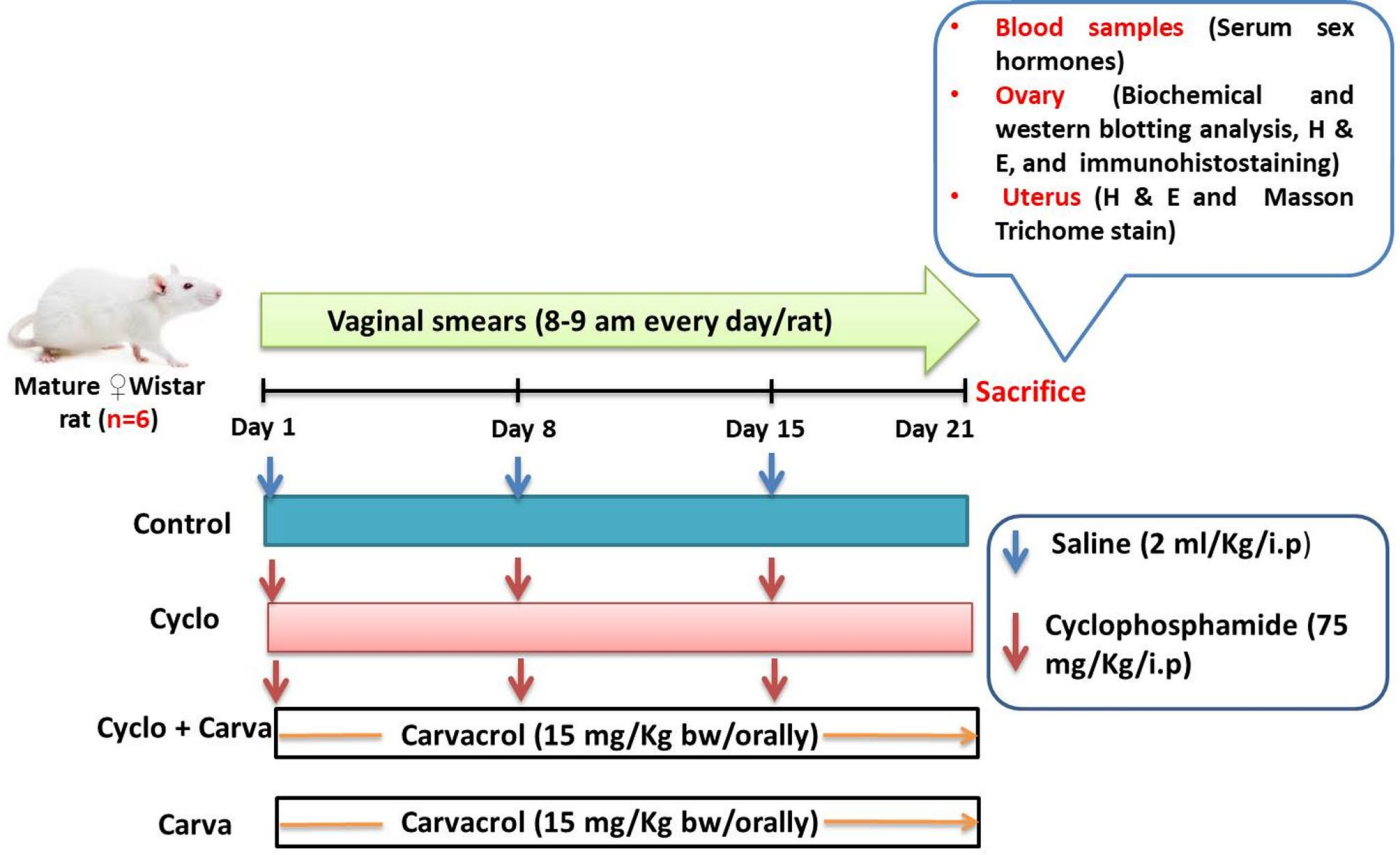
Schematic diagram of the experimental design

After the acclimation period of rats for 7 consecutive days, the animals were randomly grouped into four groups with 6 rats per group (Fig. 1). *Group I (Control)*: rats received intraperitoneal injections of saline (2 mL/Kg bw) once week/3 weeks; *Group II (Cyclo)*: rats were intraperitoneally injected with Cyclo at a dose of 75 mg/kg bw/once week/3consecutive weeks [27]; *Group III (Cyclo + Carva)*: in this group, animals received Cyclo as in group II and a daily oral dose of Carva at a dose of 15 mg/kg/bw for 21 consecutive days [28]; *Group IV (Carva)*: rats were given a daily oral dose of Carva as in the third group.

At the end of the experiment (day 21) and after the last dose of drug administration (2 h), the rats were weighed for the calculation of their final body weight. Blood samples were collected by heart puncture under anesthesia using sodium phenobarbital (40–50 mg/kg, i.p). Serum samples were separated by centrifugation at 10,000 rpm for 15 min, and the samples were kept at -80 °C to measure serum sex hormones. Upon completion of the experiment, we humanely euthanized all rats. Throughout the trial, no animals died early or before they reached a human endpoint.

Subsequently, the ovarian tissues and uterus were collected. The ovarian tissues were then promptly dissected into two sections. One section was preserved in a 10% formalin solution for histopathological and immunohistochemical examinations, while the other portion was stored in a refrigerator at -80 °C for biochemical measurements and western blotting analysis. The uterus tissue was preserved in 10% formalin solution to be histopathologically examined, followed by Masson Trichome staining to inspect the incidence of uterus fibrosis.

Furthermore, the weight of the reproductive tract was measured for every animal in the groups under study.

#### Biochemical evaluations

##### Determination of serum sex hormones

The concentrations of AMH catalogue no. (E-EL-R3022), estradiol catalogue no. (EK7003), total estrogen catalogue no. (K4266), progesterone catalogue no. (NBP2-60127), follicle-stimulating hormone (FSH) catalogue no. (KA2330), and luteinizing hormone (LH) catalogue no. (NBP2-61257), were determined using rat enzyme-linked immune sorbent assay (ELISA) kits supplied by Elabscience (USA), Boster Biological Technology (CA, USA), BioVision (CA, USA), Novus biological (USA), Abnova (Taipei City, Taiwan), Novus biological (USA), respectively. Where, six samples per group were randomly selected to measure theses parameters.

##### Ovarian tissue homogenate preparation

Using an ultrasonic homogenizer, 10% of ovarian tissue homogenate was produced in a 0.05 M phosphate buffer (pH 7) and then centrifuged in a cooling centrifuge at 4 °C to get rid of any unbroken cells, erythrocytes, nuclei, cell debris, and mitochondria. The resultant supernatant (cytoplasmic fraction) was stored at -80 °C for biochemical assessments. The protein content was estimated in tissue homogenate using a protein estimation kit (Genei, Bangalore) based on the Bradford method [29].

##### Quantification of ovarian oxidative stress indicators

The antioxidant activity was evaluated by measuring reduced glutathione (GSH), while the lipid peroxidation was indicated by measuring malondialdehyde (MDA) in ovarian tissue homogenate using rat ELISA kits acquired from Biovision, CA, USA (Catalog number #K464-100, and #K739-100, respectively). The GSH kit works by using an enzymatic cycling technique with a chromophore and GSH present. The amount of GSH present in the sample is directly proportional to the stable product that is produced by chromophore reduction and detected at 450 nm. The premise behind MDA is that it reacts with thiobarbituric acid in a sample to produce adduct that can be detected colorimetrically at 532 nm.

Where, six samples per group were randomly selected to measure theses parameters.

##### Determination of ovarian p-AKT and p-FOXO3a content

Following the manufacturer’s guidelines, the contents of p-AKT (Cat. No. SL1641Ra), and p-FOXO3a (Cat. No. MBs2615440) were quantified using rat ELISA kits purchased from SunLong Biotech Co., LTD, China, and MyBioSource, Inc. San Diego, CA, USA, respectively. The data are presented as pg/mg protein. Where, six samples per group were randomly selected to measure theses parameters.

### Western blotting analysis

The ReadyPrepTM protein extraction kit from Bio-Rad Inc. (Catalog #163–2086) was used to extract the protein from each ovarian sample. Where, three samples per group were randomly selected to measure p-PTEN (Ser380) and p-PI3K p85 alpha (Tyr580) protein expression. The Bradford Protein Assay Kit (SK3041), available from Bio Basic Inc. (Markham, Ontario, L3R 8T4 Canada), was used to quantitatively assess the extracted protein content. 20 µg protein concentration of each sample was then loaded with an equal volume of 2x Laemmli sample buffer containing 4% SDS, 10% 2-mercaptoehtanol, 20% glycerol, 0.004% bromophenol blue and 0.125 M Tris HCl. The pH was checked and brought to 6.8. The proteins in each mixture were denatured by boiling for five minutes at 95 °C before they were loaded onto a polyacrylamide gel electrophoresis using the TGX Stain-FreeTM FastCastTM Acrylamide Kit (SDS-PAGE, Bio-Rad Laboratories Inc., Cat # 161–0181). The SDS-PAGE TGX Stain-Free FastCast was prepared according to manufacture instructions. The gel was assembled in transfer sandwich as following from below to above (filter paper, PVDF membrane, gel and filter paper). The sandwich was placed in the transfer tank with 1x transfer buffer, which is composed of 25 mM Tris and 190 mM glycine and 20% methanol. Then, the blot was run for 7 min at 25 V to allow protein bands transfer from gel to membrane using BioRad Trans-Blot Turbo. The membrane was blocked in tris-buffered saline with Tween 20 (TBST) buffer and 3% bovine serum albumin (BSA) at room temperature for 1 h. The components of blocking buffer were as follow; 20 mM Tris pH 7.5, 150 mM NaCl, 0.1% Tween 20 and 3% bovine serum albumin (BSA). The primary antibodies of p-PTEN (Ser380) (Catalogue number: #9551, Cell Signaling Technology Company) and p-PI3K p85 alpha (Tyr580) (Catalogue number: #AF4370, Affinity Biosciences Company) were diluted in TBST following the manufacturer guidelines. Incubation was done overnight in each primary antibody solution, against the blotted target protein, at 4 °C. The blot was rinsed 3–5 times for 5 min with TBST. Incubation was done in the HRP-conjugated secondary antibody (Goat anti-rabbit IgG- HRP-1 mg Goat mab -Novus Biologicals) solution against the blotted target protein for 1 h at room temperature. The blot was rinsed 3–5 times for 5 min with TBST. The chemiluminescent substrate (Clarity TM Western ECL substrate Bio-Rad cat#170–5060) was applied to the blot according to the manufacturer’s recommendation. Briefly, equal volumes were added from solution A (Clarity western luminal/enhancer solution) and solution B (peroxidase solution). The chemiluminescent signals were captured using a CCD camera-based imager. Image analysis software was used to read the band intensity of the target proteins against control sample beta actin (housekeeping protein) by protein normalization on the ChemiDoc MP imager.

### Identification of vaginal cytology

Concerning the identification of vaginal cytology, vaginal smears were taken once a day between 8 and 9 in the morning to monitor the estrous cycle’s advancement during the experiment. Quickly and gently, sterile cotton-tipped swabs dampened with distilled water were inserted into the vaginal opening; the entrance was made relatively shallow, about 1 cm, to prevent excessive cervical stimulation and the ensuing pseudo-pregnancy [30]. They were then turned (one twist) against the vaginal wall with extreme caution. Rats were not given anesthesia to collect smears. The obtained sample of vaginal epithelial cells was then put on glass slides and dried at 37 °C. As soon as the smears were ready and dried, they were quickly fixed by dipping them three to five times in a 70% alcohol container. The slides were then immediately stained with Methylene blue stain. The slides were stained with methylene blue, washed with tap water, and then analyzed. The slides may be inspected while still wet, or a cover slip might be used [31, 32]. The stage of the estrous cycle was determined as follows [33]: Metestrus (equal percentage of nucleated epithelial cells, cornified epithelial cells, and leukocytes) and diestrus (dominant leukocytes) differ from proestrus (nucleated epithelial cells) and estrous (cornified epithelial cells).

### Histopathological examination

Tissue samples (ovaries and uterus) were preserved in 10% formalin for 48 h, after being dehydrated in an increasing concentration of alcohol (from 70 to 100%) for 2 h in each, the sample was after wards clarified in xylene alcohol (50:50), exelon (three times) for 30 min., and embedded in paraffin wax (at 58 °C for 45 min.) to obtain paraffin blocks for light microscopic analysis. Specimens were sectioned into 4–5 μm thickness using rotary microtome, and arranged on slides. Following these components’ deparaffinization and production of an increasing grade of alcohol (70, 89, 90, 100%, 30 min for each step),

(100–20%), stained with Hematoxylin and Eosin (H&E) and subjected to microscopic examination [34]. A Leica UA510CA light microscope and a Leica Q-Win image analysis system (Leica Micros Imaging Solutions Ltd., Cambridge, UK) were used to investigate the tissue samples.

Using Masson’s trichrome staining, the fibrotic alterations and collagen deposition of uterus tissues were observed and examined [35]. Collagen density (Area %) in eight different fields per group was quantified using tissue sections stained using MT stain [36]. The principle behind quantifying collagen in uterus tissues stained with MT stain using the Colour Deconvolution plugin in Image J is based on the separation of different colour channels in the stained tissue sections to isolate the collagen fibers and then quantifying them through thresholding and image analysis [37].

### Morphometric analyses

Eight different non-overlapping fields were taken for image analysis at magnification x200. The number of ovarian follicles was measured with the determination of the type of follicles as presented in Table 1 [38], using the imaging analysis of a Lecia Qwin 500 Image Analyzer (Lecia Imaging Systems Ltd, Cambridge, UK).

**Table 1:** Classification criteria of follicles

Follicle	Morphologic characteristics of cells
Primordial follicle	Oocyte partially or completely encapsulated by squamous pregranulosa cells
Primary follicle	Granulosa cells (single layer) show enlargement
Secondary follicle (Preantral follicle)	Oocyte encapsulated by more than 2 layers of granulosa cells; no antrum formation
Tertiary (Antral)	Oocyte encapsulated by more than 2 layers of granulosa cells with antrum

### Immunohistochemistry study

Following the protocol described previously [39], the immunostaining analysis was performed. Sections obtained were maintained in an incubator at 37 °C overnight. Deparaffinization of tissue sections using various methods. First, the sections were left in xylol for 5 min and then dehydrated in a graded alcohol series for 3 min each. The sections were then fixed in EDTA buffer in a microwave for 10 min to remove formaldehyde. After cooling to 25 °C, tissues were circled with a Pap pen, washed with PBS three times, and the sections rehydrated and exposed to antigen unmasking in 0.1 mol Tris–HCl buffer l–1 (pH 10) with 5% (w/v) urea by heating for 20 min in a Sharp 1000 W microwave oven for 0.1 mg proteinase K ml–1 (Sigma) in 0.1 mol Tris–EDTA buffer l–1 (pH 7.4). To recover the antigenicity, the sections were further treated with a heated antigen retrieval solution that contained a citrate antigen retrieval solution (Beyotime, China). The samples were blocked by 10% goat serum/0.3% Triton X-100 in PBS for 30 min. Following this, they were incubated for an hour at room temperature with primary anti-Caspase-3 (sc-7272, diluted 1:200, Sanat Cruz, Biotechnology, Inc.) and p-AKT (66444-1-Ig, diluted 1:300, Proteintech, Germany). Following that, the sections were cleaned and probed with anti-mouse/rabbit second antibodies labeled with horseradish peroxidase. A diaminobenzidine substrate kit (BioSB, USA) was utilized to create a peroxidase substrate. The slides underwent a sequence of ethanol grades for dehydration, hematoxylin counterstaining, and Permount Mounting Medium (Specimens of Harbin Green Technology Development Co., China) mounting. Negative control slides were obtained by escaping the incubation with the primary antibody step.

Using images of immunostained slides, the intensity of caspase 3 and p-AKT immunopositive cells was estimated as an area percentage (%) across eight different fields. The expression of caspase 3, and p-AKT was scored for percentage and intensity of immunostaining as follows: low for staining less than 10% of ovarian tissue cells, mild for staining 10–25%, moderate for staining 25–40%, moderate to high for staining 40–65%, a high expression for staining in more than 65% [40, 41].

### Statistical analyses

Statistical analyses were performed using GraphPad Prism software (version 6.0 for Windows, GraphPad Inc., San Diego, CA). The Shapiro-Wilk test was used to verify the data’s normality at *p* > 0.05. The Student “T” test was used to examine changes in the rat’s final body weight relative to its initial body weight. Group differences were evaluated using a one-way ANOVA. In case of normality of data, Tukey’s post-hoc test was applied to control for Type I error across multiple comparisons. Meanwhile, the Kruskal–Wallis test followed by Dunn’s multiple comparisons test was applied in case of non-normality data. Exact two-sided P values are reported to three or four decimals. Standardized effect sizes are presented as η² (R^2^) for omnibus ANOVAs. 95% confidence intervals (95% CI) were calculated for mean differences and effect sizes.

## Results

### Effect of carvacrol treatment on body weight and reproductive tract weight

**Fig. 2:**
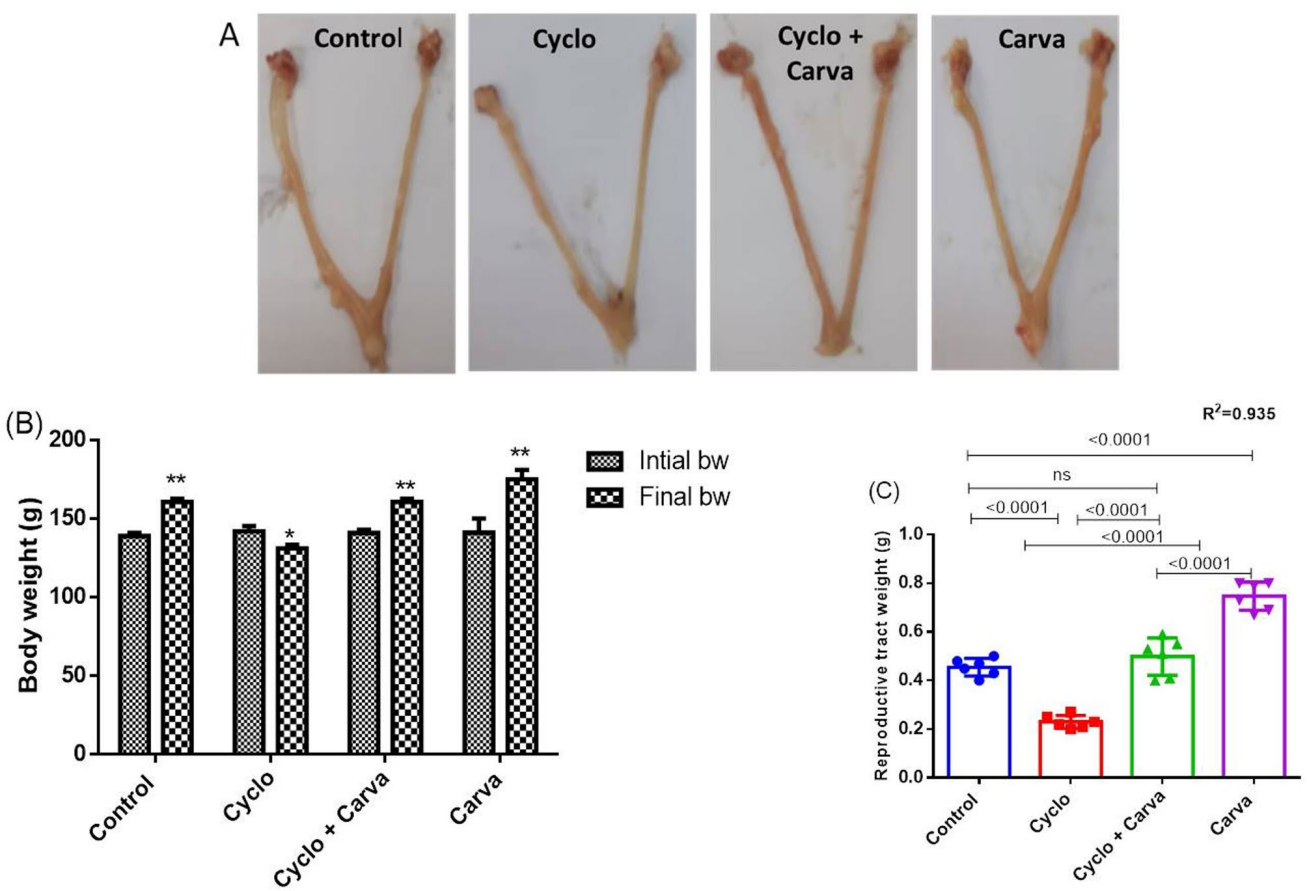
Photographs showing the genital tract dissected from the studied groups (**A**). Effect of carvacrol treatment on changes of body weight (**B**) and reproductive tract weight (**C**) in cyclophosphamide-induced ovarian toxicity in rats. Values are stated as mean ± CI (*n* = 6). Student “T” test was applied to compare the final body weight relative to their initials for each group. *^,^**: Denote significance difference at *p* < 0.05 or 0.001 respectively. One way ANOVA followed by Tukey’s test for multiple comparisons was applied. Carva; carvacrol, Cyclo; cyclophosphamide

Concerning their initial bw, rats intoxicated with Cyclo manifested a significant decline in their bw by 7.29%. Unlike, oral administration of Carva at a dose of 15 mg/kg bw alone or along with Cyclo showed a marked increase in final bw by 24.08% and 14.07%, respectively, relative to their initials (Fig. 2B). Similarly, a significant decrease (49.54%) in reproductive tract weight was documented in the Cyclo-challenged group, matched to those of saline-injected rats. Conversely, concurrent treatment of Carva with Cyclo markedly normalized the weight of rats’ reproductive tract as those of control. It is noted that a significant rise in reproductive tract weight was recorded in rats treated only with Carva compared to the control group (Fig. 2C). Moreover, Fig. 2A represents the morphology of rats’ genital tracts in the different studied groups.

### Effects of carvacrol treatment on serum sex-hormonal status

As depicted in Table 2, significant repressions (*p* value < 0.0001) in serum AMH (59.09%), estradiol (72.56%), progesterone (69.10%), and total estrogen (59.44%) levels, along with a marked increase in serum FSH (1.81 fold) and LH (2.98 fold) concentrations were observed in Cyclo-challenged rats, in comparison to the negative control animals. Conversely, Cyclo-injected rats orally treated with Carva at a dose of 15 mg/kg bw significantly (*p* value < 0.0001) improved the serum levels of the aforementioned hormones, as reported by the elevation of AMH (77.67%), estradiol (2.55 fold), progesterone (2.33 fold), and total estrogen (59.24%) levels, as well as diminishing serum FSH (34.33%) and LH (38.27%) concentrations, corresponding to the Cyclo-intoxicated rats. Interestingly, non-significant changes (*p* value > 0.05) were observed regarding these hormones in the Carva-treated group, compared to the control group.

**Table 2:** Effects of carvacrol treatment on serum sex-hormonal status

Groups	AMH (pg/ml)	Estradiol (pg/ml)	Progesterone (ng/ml)	Total estrogen (ng/ml)	FSH (ng/ml)	LH (mIU/ml)
Control	222.48 ± 3.53 ^a^	99.77 ± 4.18 ^a^	6.57 ± 0.08 ^a^	48.27 ± 1.13 ^a^	2.03 ± 0.03 ^a^	20.36 ± 0.78 ^a^
Cyclo	91.02 ± 5.10 ^b^	27.38 ± 1.34 ^b^	2.03 ± 0.07 ^b^	19.58 ± 0.62 ^b^	3.67 ± 0.05 ^b^	60.62 ± 2.16 ^b^
Cyclo + Carva	161.72 ± 3.77 ^c^	69.87 ± 1.40 ^c^	4.73 ± 0.28 ^c^	31.18 ± 1.22 ^c^	2.41 ± 0.07 ^c^	37.42 ± 1.00 ^c^
Carva	217.75 ± 3.41 ^a^	95.65 ± 3.32 ^a^	6.38 ± 0.15 ^a^	46.15 ± 1.33 ^a^	1.97 ± 0.06 ^a^	18.86 ± 0.70 ^a^
η²	0.972	0.954	0.959	0.957	0.969	0.971
F-ratio *P* <	233.91 0.001	137.34 0.001	157.76 0.001	148.46 0.001	208.46 0.001	233.29 0.001

Values are presented as mean ± SE ( *n* = 6). Columns with disparate superscripts (a, b, c) are significantly different at *p* < 0.001 (Tukey’s test). *Carva* carvacrol, *Cyclo* cyclophosphamide.

###  Effect of carvacrol treatment on ovary oxidative stress indicators 

**Fig. 3:**
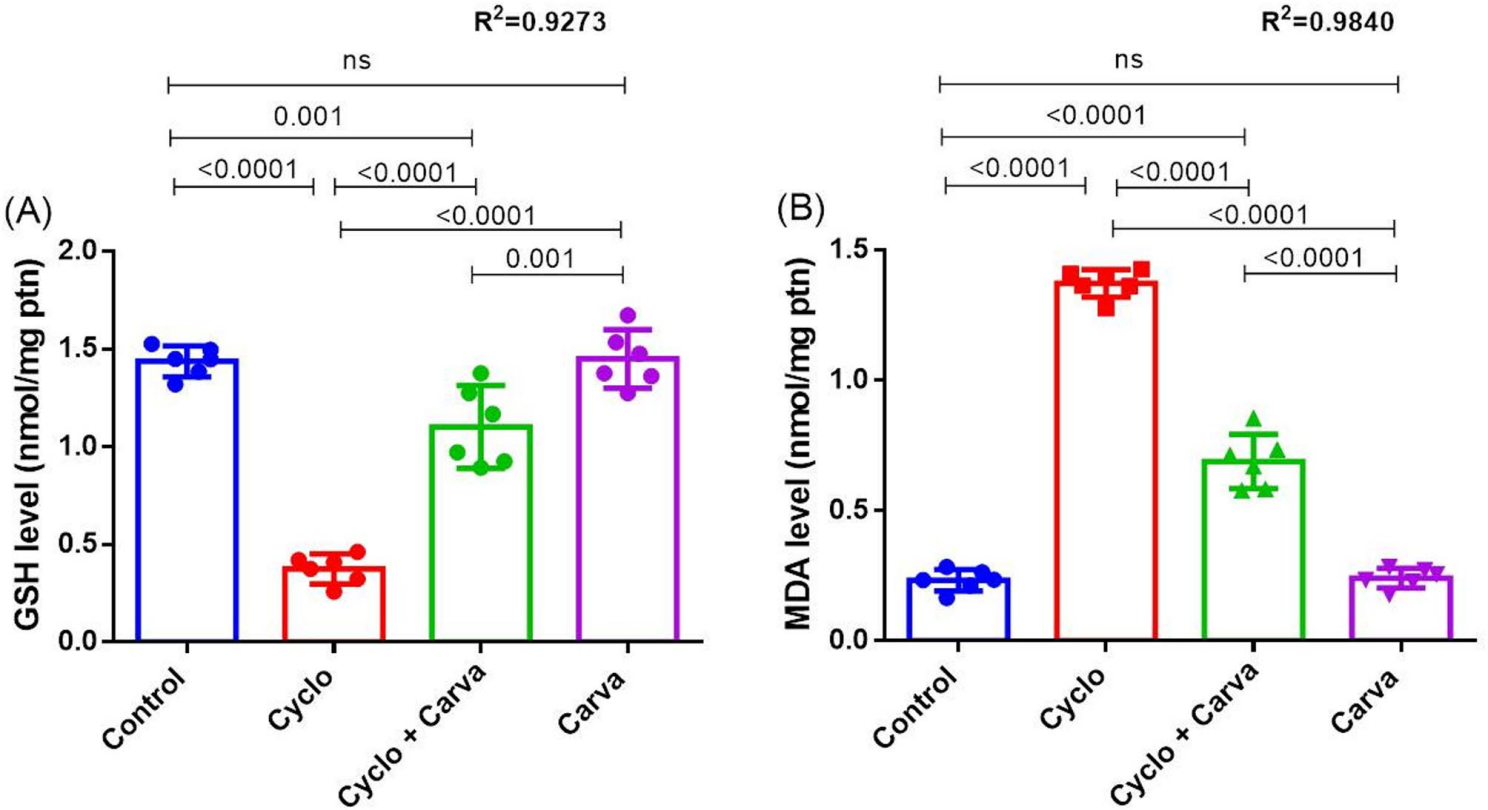
Effect of carvacrol treatment on ovary oxidative stress indicators; GSH (**A**) and MDA (**B**) levels in cyclophosphamide-induced ovarian toxicity in rats. Values are stated as mean ± CI (*n* = 6). One way ANOVA followed by Tukey’s test for multiple comparisons was applied. Carva; carvacrol, Cyclo; cyclophosphamide

A single intraperitoneal injection of Cyclo at a dose of 75 mg/kg bw/week for 3 consecutive weeks resulted in a significant decline in GSH level of 73.88% (p value < 0.0001), in combination with a boost in MDA content of 5.89 fold (p value < 0.0001) in rat ovarian tissue homogenate relative to the control rats, reflecting the incidence of oxidative stress following Cyclo exposure (Fig. 3).

Contrary to that, concomitant administration of Carva with Cyclo remarkably (p value < 0.0001) alleviated the imbalance of the oxidant/antioxidant status of ovarian tissue, as demonstrated by amplifying the GSH level by 2.94 fold and lessening the MDA content by 49.17%, in comparison to the Cyclo-intoxicated group. It is worth mentioning that Carva treatment per se did not show any significant alterations in these parameters, compared to the control rats (Fig. 3).

### Effects of carvacrol treatment on ovarian p-PI3K/p-PTEN/p-AKT/p-FOXO3a axis

**Fig. 4:**
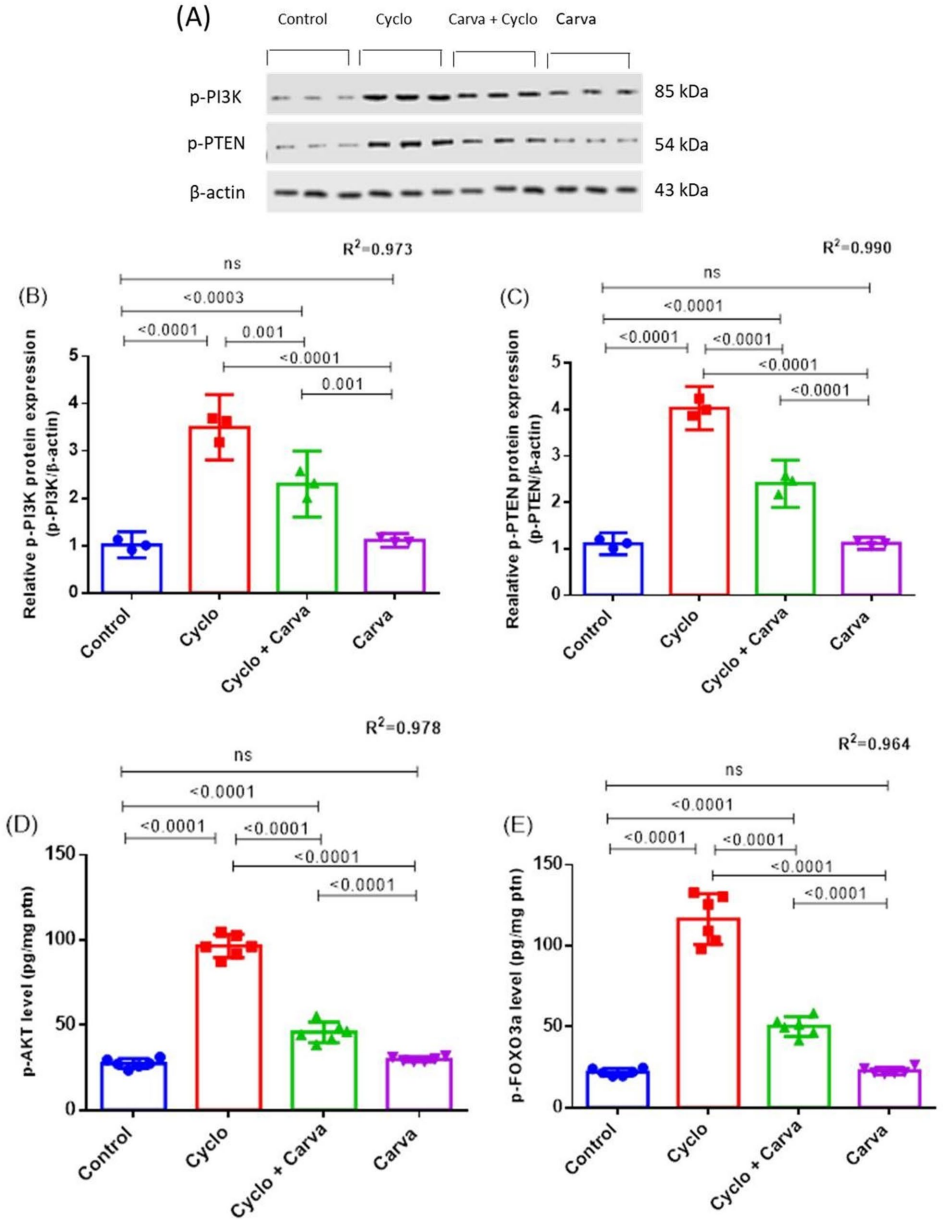
Western blots image of p-PI3K, p-PTEN, and β-actin proteins (**A**). Western blotting analysis of relative protein expression of the ovarian p-PI3K (**B**) and p-PTEN (**C**) levels, as well as the protein content of ovarian p-AKT (**D**) and p-FOXO3a (**E**) in the studied groups. Values are stated as mean ± CI (*n* = 6). For western blotting analyses, *n* = 3 independent samples per group. One way ANOVA followed by Tukey’s test for multiple comparisons was applied. Carva; carvacrol, Cyclo; cyclophosphamide

Corresponding to the control group, a remarkable upregulation in the levels of p-PI3K and p-PTEN protein expression by 4.03 fold and 3.50 fold respectively, along with a significant rise in p-AKT (251.26%) and p-FOXO3a (3.51 fold) content were observed in ovarian tissue of Cyclo-challenged rats (Fig. 4). On the other side, oral administration of Carva at a dose of 15 mg/kg bw to Cyclo-intoxicated animals significantly antagonized the increase in these markers as shown by down-regulating the expression of p-PI3K and p-PTEN proteins and suppressing p-AKT (52.58%) and p-FOXO3a (56.95%) levels, matched to the Cyclo-model group. Furthermore, no marked variations in these biomarkers were found in the Carva-treated group in comparison to the normal rats as illustrated in Fig. 4.

### Identification of vaginal cytology

**Fig. 5:**
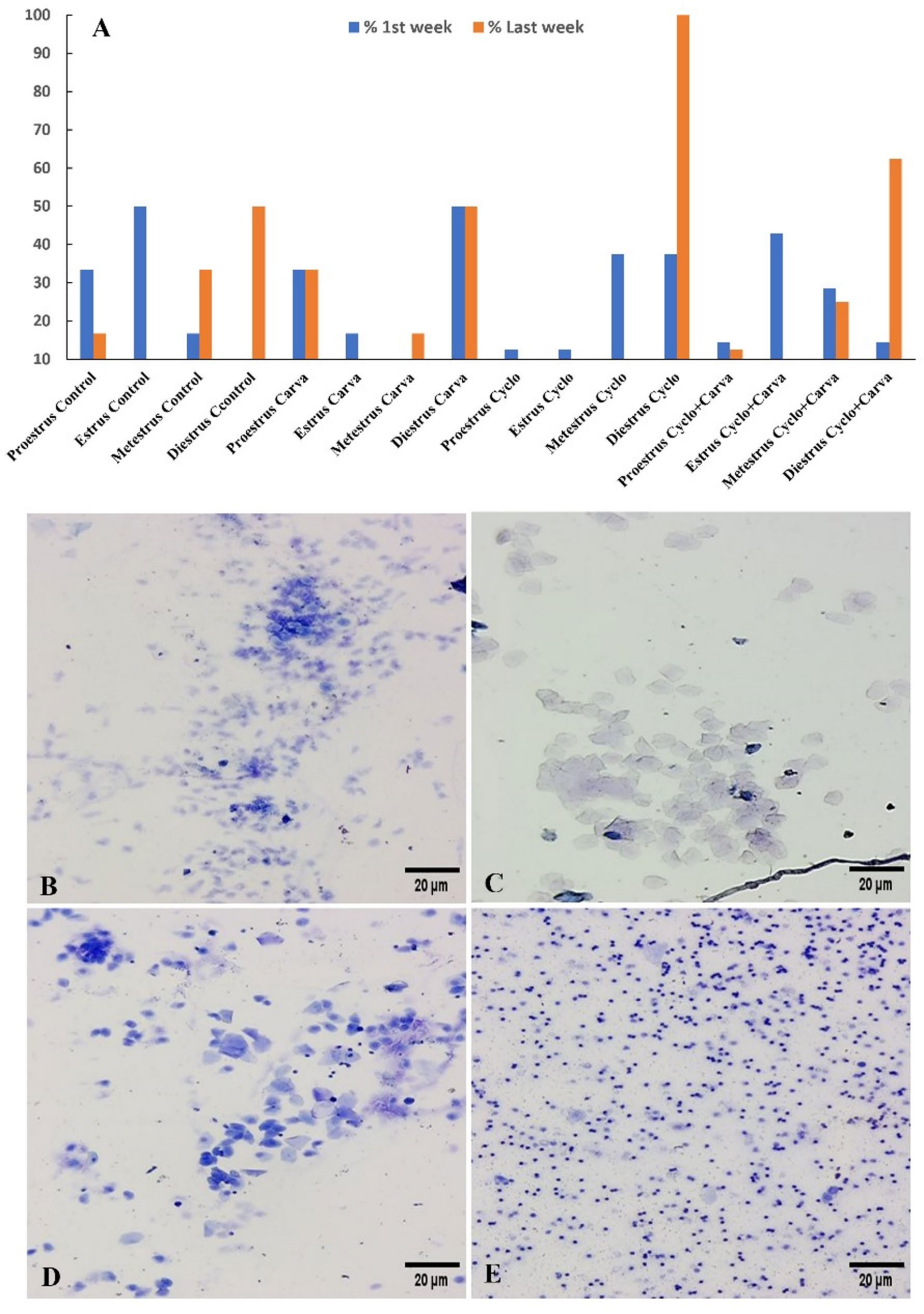
Identification of vaginal cytology and determination of each estrous cycle stage. **A** Percentage of animals/group came in each estrous phase at the 1st week and last week of the experiment. **B** Predominately composed of nucleated epithelial cells during proestrus.** C** Clusters of cornified epithelial cells predominate during estrus.** D** The same ratio of leukocytes to nucleated and cornified epithelial cells occurs during metestrus.** E** During Diestrus, leukocytes predominated in large quantities. (B, C, D & E, Methylene blue staining, scale bar 20 μm). Carva; carvacrol, Cyclo; cyclophosphamide

The percentage of rats per group that came in each phase of the estrous cycle at the first and last week of the experiment is shown in Fig. 5A. Our results revealed that both the average length of the estrous cycle and the state of the persistent di-estrus were increased by Cyclo. In contrast, the length of the entire cycle was regularized by co-treatment with Carva. An observable reduction in ovarian reserve was brought on by Cyclo, as seen by an extended and erratic menstrual cycle. The Cyclo-treated group’s estrous cycle was extended by up to 8–10 days, according to the obtained data. About 60.00% of the rats in the Cyclo + Carva-treated group went back to having their normal estrus cycles, and they also displayed shorter estrus cycles than the Cyclo group, lasting between 5 and 6 days. While Fig. 5B, C, D & E depicted the features of different estrous cycle stages using methylene blue staining.

### Histopathological findings

#### Haematoxylin and Eosin staining

##### Ovary

**Fig. 6:**
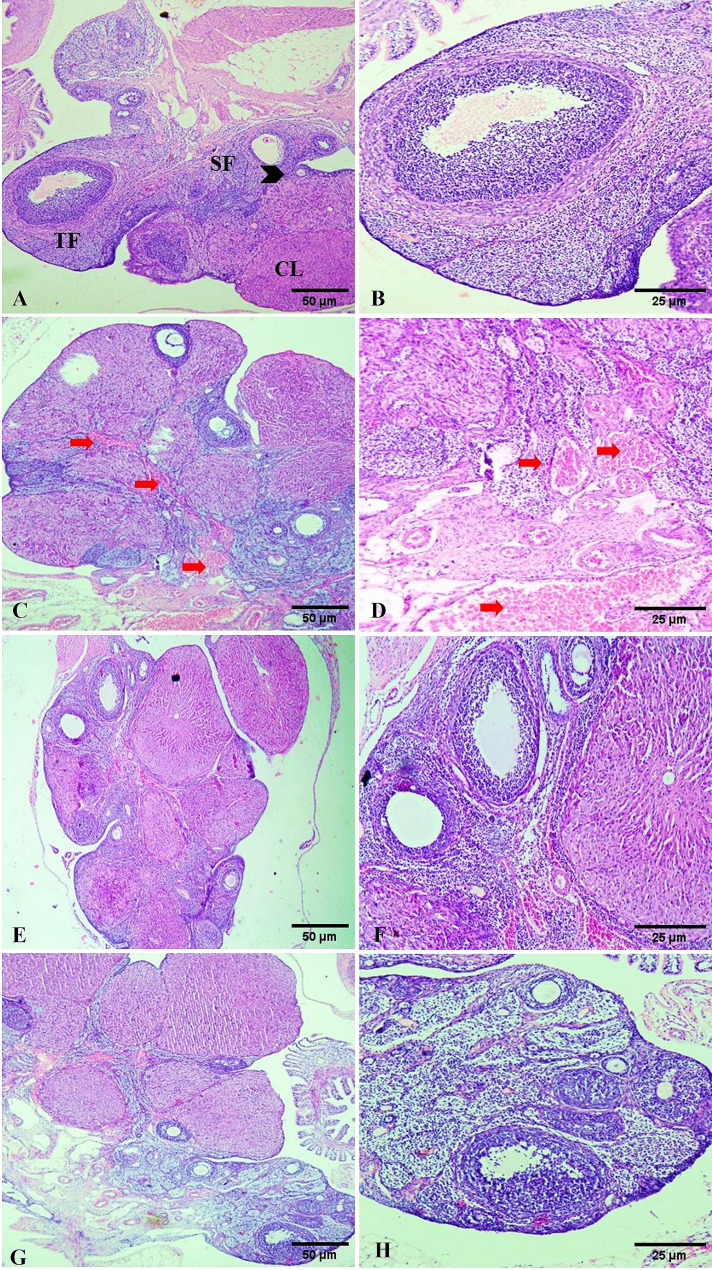
Photomicrograph of ovarian tissue of control rats (**A**,** B**) showing primary follicle (Arrowhead), secondary follicle (SF), tertiary follicle (TF), and corpus luteum (CL). Cyclo treated rats showing moderate ovarian venous congestion and hemorrhages (Red arrows) especially around CL with severe degenerative changes and follicular atresia **(C**,** D**). Cyclo + Carva treated rats showing a remarkable improvement in the histologic appearance with multiple mature follicles as well as CL-associated and decreased vascular congestion (**E**,** F**). Carva treated rats (**G**,** H**) showed normal ovarian structures with mild venous congestion (H&E Scale bar 25, 50). Carva; carvacrol, Cyclo; cyclophosphamide

The primordial follicle, primary, secondary, and multiple mature Graafian follicles, along with Corpora lutea (CL), displayed normal histologic appearances at different stages of development and maturation, according to the histopathological examination of the ovarian sections of control rats (Fig. 6A, B). In contrast, photomicrographs of the ovarian sections of rats given Cyclo showed atrophied ovarian tissue along with vascular congestion, ovarian stroma hemorrhage, and hemorrhage surrounding the CL (Fig. 6C, D). A marked improvement in the ovarian tissue of the Cyclo + Carva-treated group has been observed, as indicated by the existence of many mature follicles, decreased vascular congestion, and CL-associated growth (Fig. 6E, F). The rats that were given Carva displayed a typical histological architecture with a minimal level of vascular congestion (Fig. 6G, H).

##### Uterus

**Fig. 7:**
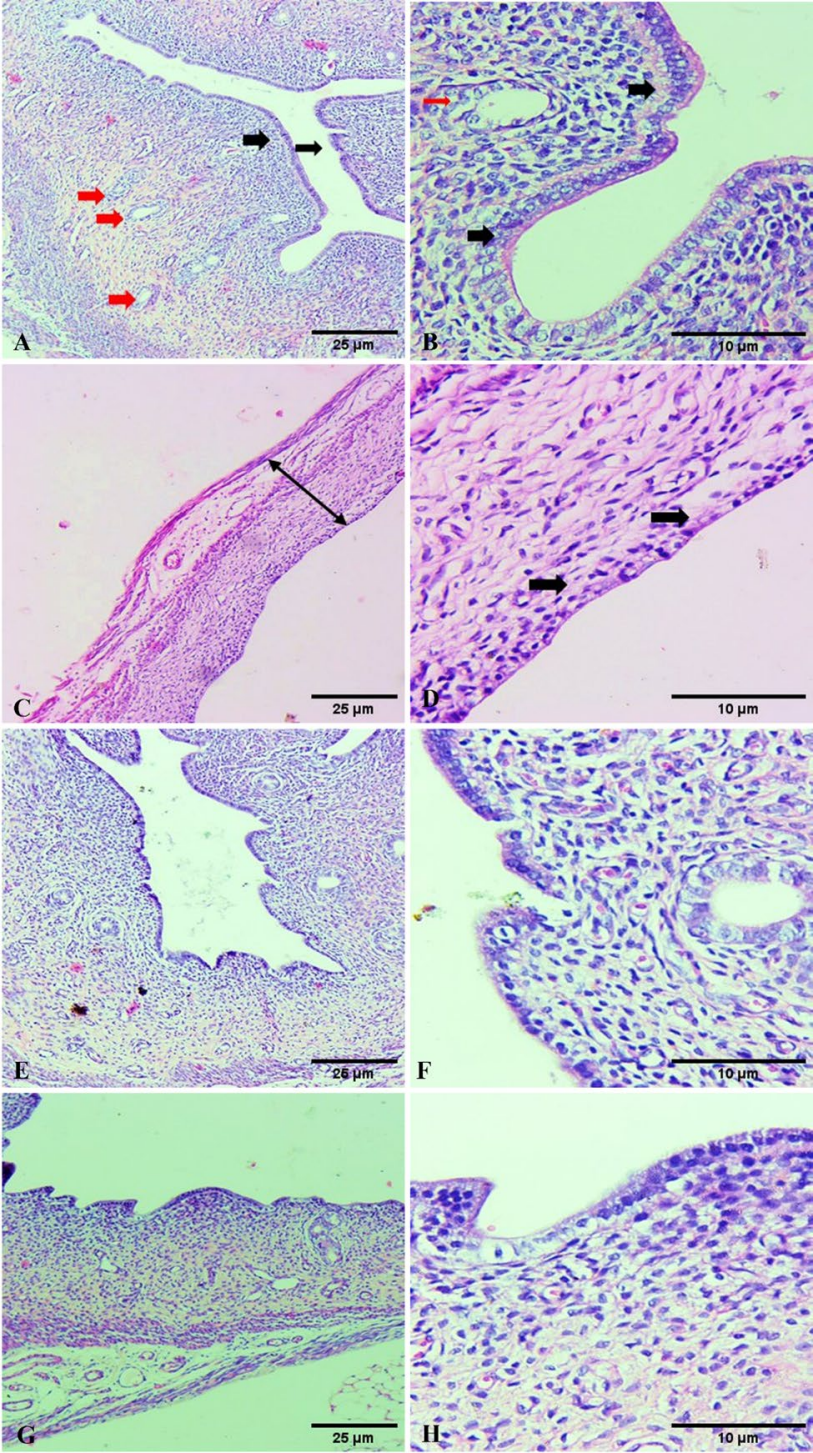
Photomicrograph of uterine tissue of control rats (**A**,** B**) showing the presence of several proliferating endometrial glands surrounded by stroma (Red arrows), together with intact non-degenerated endometrial lining (Black arrows). Cyclo treated rats showed a severe decrease in the thickness of the uterine wall (Double arrow), a severe decline in number of the uterine glands, and decreased glandular proliferation associated with inactive cuboidal lining endometrial epithelium (Black arrows) (**C**,** D**). Cyclo + Carva treated rats showed restoration of the normal uterine histologic structures with the presence of multiple uterine glands (**E**,** F**). Rats given Carva treatment exhibited intact, non-degenerated endometrial lining and many, growing endometrial glands encircled by stroma. (**G**,** H**) (H&E Scale bar 10, 25 μm). Carva; carvacrol, Cyclo; cyclophosphamide

Examined uterine slices from control rats showed an intact, non-degenerated endometrial lining, many proliferating endometrial glands surrounded by stroma, and a normal histological architecture (Fig. 7A, B). Inactive low cuboidal to the flattened epithelial lining with decreased inactive endometrial glands, fibrotic stroma, decreased glandular proliferation, and decreased uterine diameter were all observed in the uterine tissue sections of rats treated with cyclo. There was also degeneration and vacuolization in epithelial cells (Fig. 7C, D). Multiple uterine glands and the restoration of normal uterine histologic features were observed in the treatment group that received Cyclo + Carva (Fig. 7E, F). Rats given Carva treatment exhibited an intact, non-degenerated endometrial lining and many growing endometrial glands encircled by stroma (Fig. 7G, H).

#### Masson’s trichrome

**Fig. 8:**
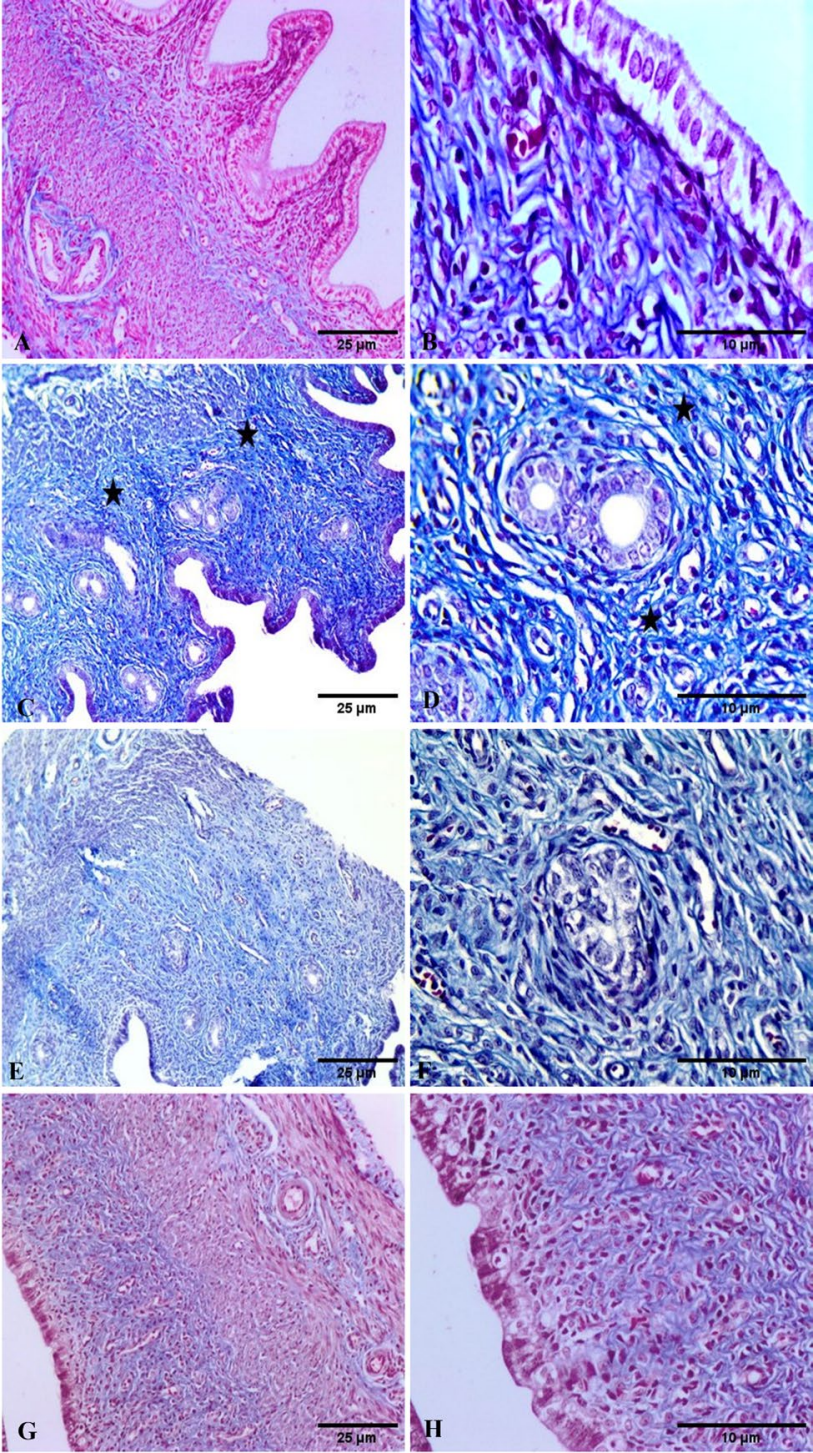
Photomicrograph of uterine tissue of control rats (**A**,** B**) showed normal uterine architecture with the presence of mild deposition of collagen fibers in the endometrial stroma surrounding the uterine glands. Cyclo treated rats showed a marked increase in the blue stained collagen fibers (black star) throughout the uterine stroma with strong positive histochemical reaction for collagen fibers (**C**,** D**). Cyclo + Carva treated rats showed restoration of the normal uterine histologic structures with a decrease in the collagen fibers deposition in the endometrial stroma (**E**,** F**). Carva treated rats showed normal uterine architecture with the presence of mild deposition of collagen fibers in the endometrial stroma surrounding the uterine glands (**G**,** H**). (Masson’s trichrome H&E Scale bar 10, 25 μm). Carva; carvacrol, Cyclo; cyclophosphamide

Normal uterine architecture with mild collagen deposition in the endometrial stroma surrounding the uterine gland has been observed in the uterine tissue of control rats and carva group (Fig. 8A, B, and G, H, respectively). Following cyclo intoxication, uterine tissue parenchyma exhibited clearly defined blue-colored collagen fibers with a robust positive histo-chemical reaction for collagen fibers throughout the uterine stroma, as demonstrated in Fig. 8C, D. There was an obvious reduction in collagen deposition in the uterine tissue of Cyclo + Carva-treated rats, as indicated by a decrease in the quantity of blue-colored fibers (Fig. 8E, F). The collagen area % of the studied groups is presented in Fig. 14A.

#### Morphometric analyses

**Fig. 9:**
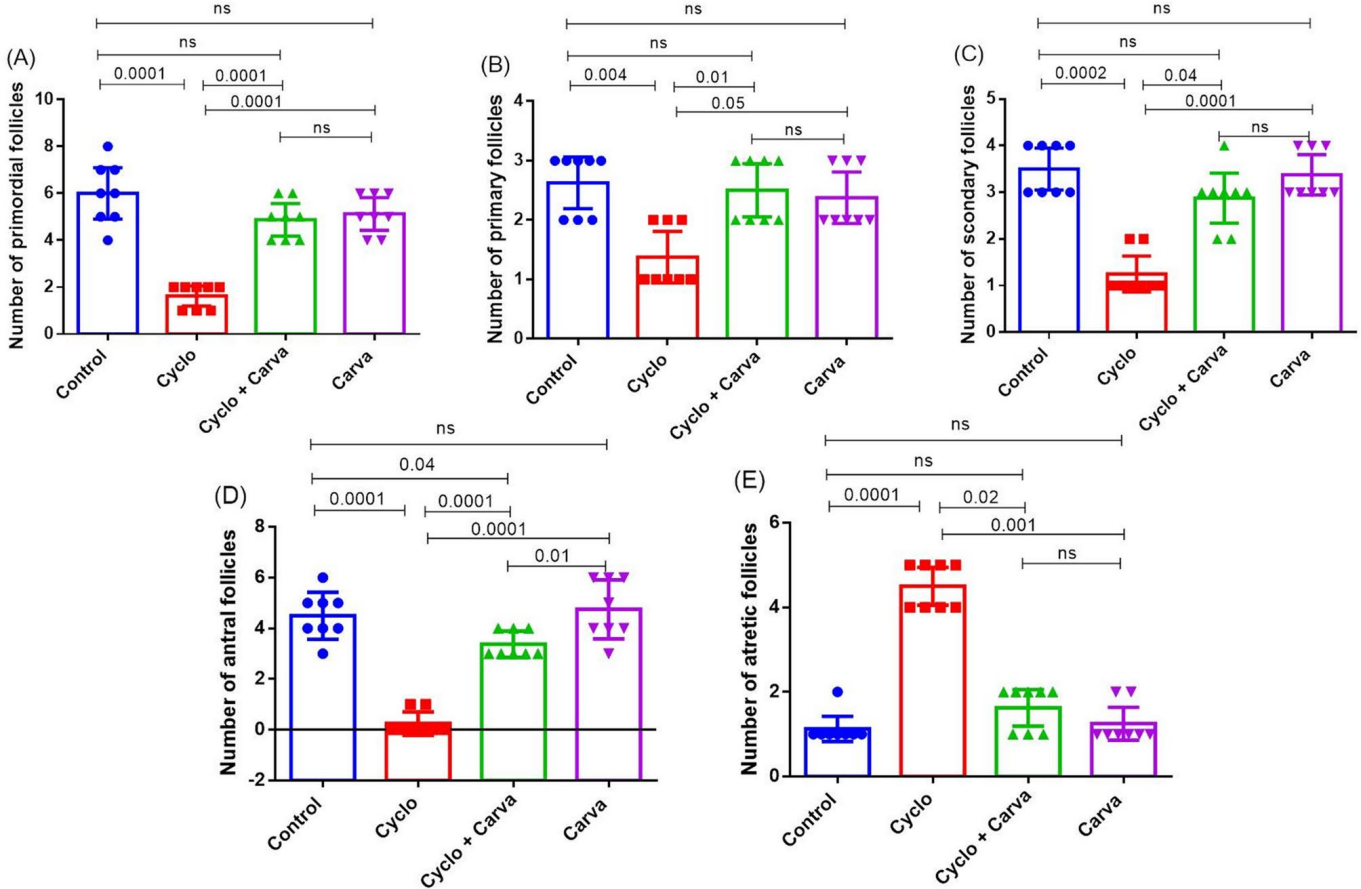
Effects of carvacrol treatment on primordial (**A**), primary (**B**), secondary (**C**), Antral (**D**), and atretic (**E**) follicle count in cyclophosphamide-induced ovarian toxicity in rats. Values are presented as mean ± CI (*n* = 8 fields). Kruskal–Wallis test followed by Dunn’s multiple comparisons test was applied. Carva; carvacrol, Cyclo; cyclophosphamide

A dramatic loss of ovarian follicle (primordial, primary, secondary, and antral) count, coupled with a significant boost in the atretic ones has been documented in the Cyclo-intoxicated rats when compared to the other groups. This significant decline underscores the impact of Cyclo treatment on ovarian follicle number and morphology, as evident from the comparative data presented in Fig. 9. By contrast, significant improvements in the number of primordial, primary, secondary, and antral ovarian follicles, along with a marked decline in the atretic follicles have been recorded in the Carva + Cyclo-treated group (Fig. 9 and supplementary file Fig. 1S).

### Immunohistochemical results

**Fig. 10:**
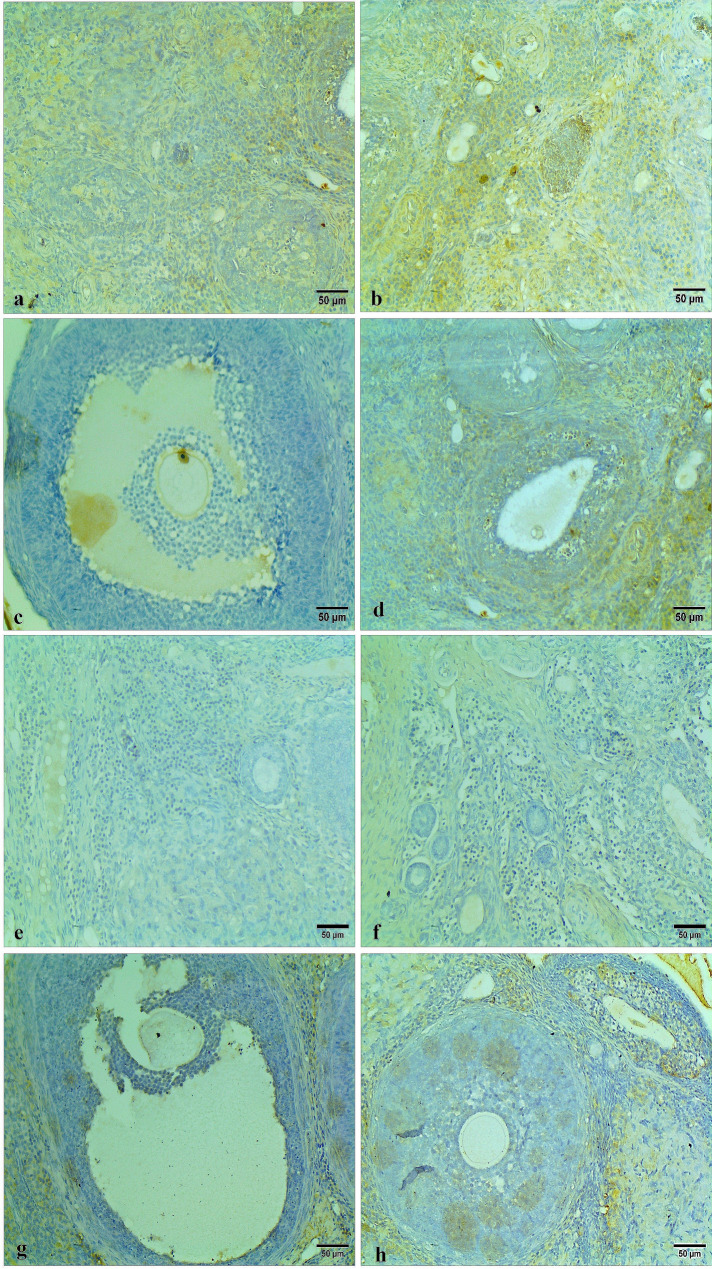
Immunohistochemical expression of caspase 3 of ovarian tissues of the control group showing low caspase 3 positivity in the apoptotic cells within the ovarian cortex (**a**), ovarian medulla (**b**), Graafian follicle (**c**), and secondary follicle (**d**). Carva-treated group showing low caspase 3 positivity in the apoptotic cells within the ovarian cortex (**e**), ovarian medulla (**f**), antral follicle (**g**), and secondary follicle (**h**). (Immunohistochemical staining Scale bar 50 μm). Carva; carvacrol

**Fig. 11:**
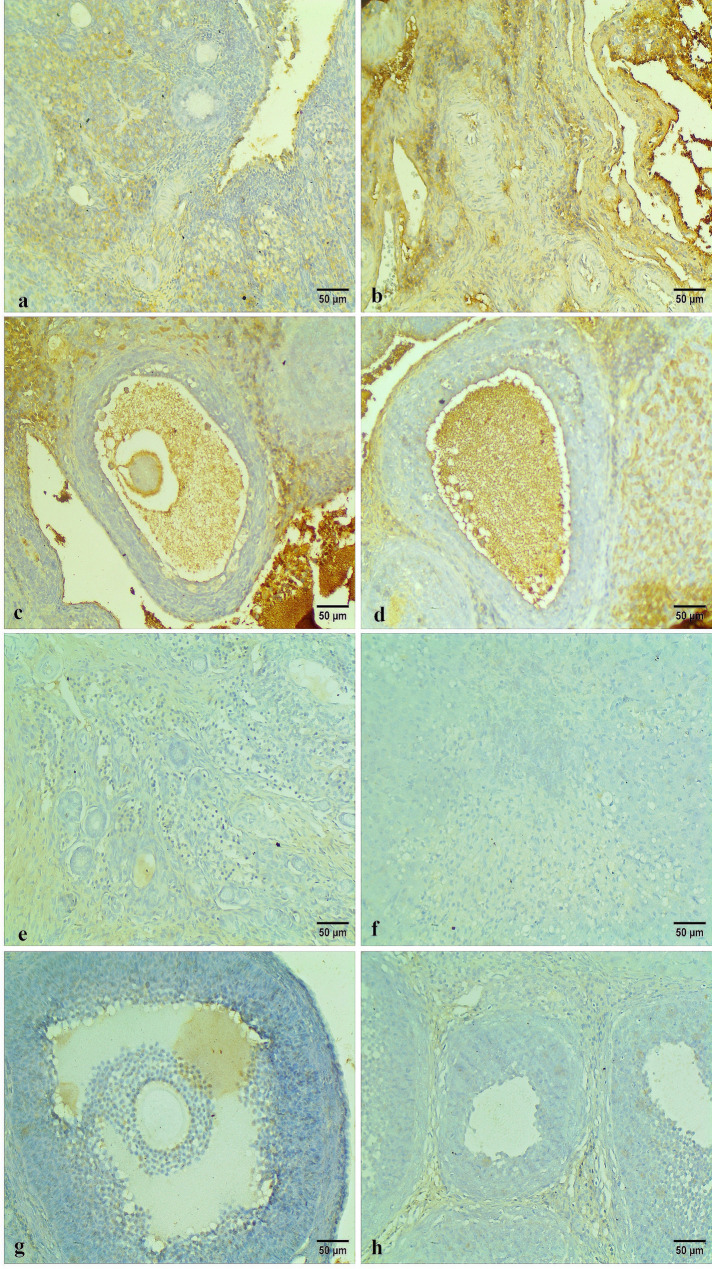
Immunohistochemical expression of caspase 3 of ovarian tissue of the Cyclo treated group showing high caspase 3 immunoreactivity in the apoptotic cells within the ovarian cortex (**a**), ovarian medulla (**b**), Graafian follicle (**c**), and secondary follicles (**d**). Cyclo + Carva treated group showing low caspase 3 positivity in the apoptotic cells within the ovarian medulla (**e**), corpus luteum (**f**), Graafian follicle (**g**), and secondary follicle (**h**). (Immunohistochemical staining Scale bar 50 μm). Carva; carvacrol, Cyclo; cyclophosphamide

The caspase 3 immunohistochemical staining appeared as intracytoplasmic, nuclear brown colorations of the ovarian follicles, cortex, medulla, and stroma. Ovarian tissues of the control group and Carva-treated rats showed low caspase 3 reactivity in the apoptotic cells, indicating minimal cellular apoptosis (Fig. 10). In contrast, the Cyclo-treated rats exhibited high caspase 3 reactivity in the apoptotic cells as compared to the other groups, reflecting severe apoptosis intensity. This heightened reactivity highlights the pronounced apoptotic effect of Cyclo on the ovarian tissues (Figs. 11a, b, c, and d). However, the ovarian tissues of Cyclo + Carva-treated rats showed considerable improvement with caspase 3 reactivity ranging from low to mild, suggesting the anti-apoptotic effect of Carva (Figs. 11e, f, g, and h). The area percentage of caspase 3 immunostaining in the different groups is represented in Fig. 14C.

**Fig. 12:**
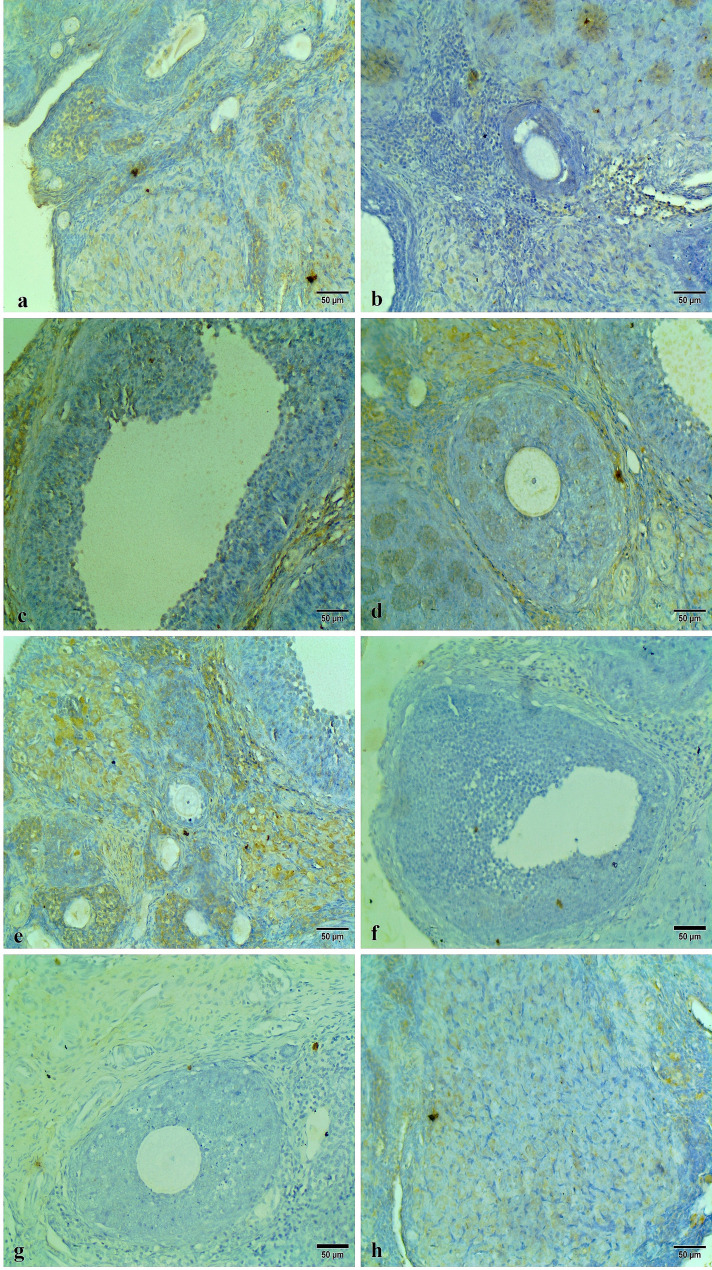
Immunohistochemical expression of p-Akt of ovarian tissues of the control group showing low p-Akt immunoreactivity within the ovarian cortex (**a**), ovarian medulla (**b**), Graafian follicle (**c**), and secondary follicle (**d**). Carva-treated group showing low p-Akt immunoreactivity within the ovarian medulla (**e**), antral follicle (**f**), secondary follicle (**g**), and corpus luteum (**h**). (Immunohistochemical staining Scale bar 50 μm). Carva; carvacrol

**Fig. 13:**
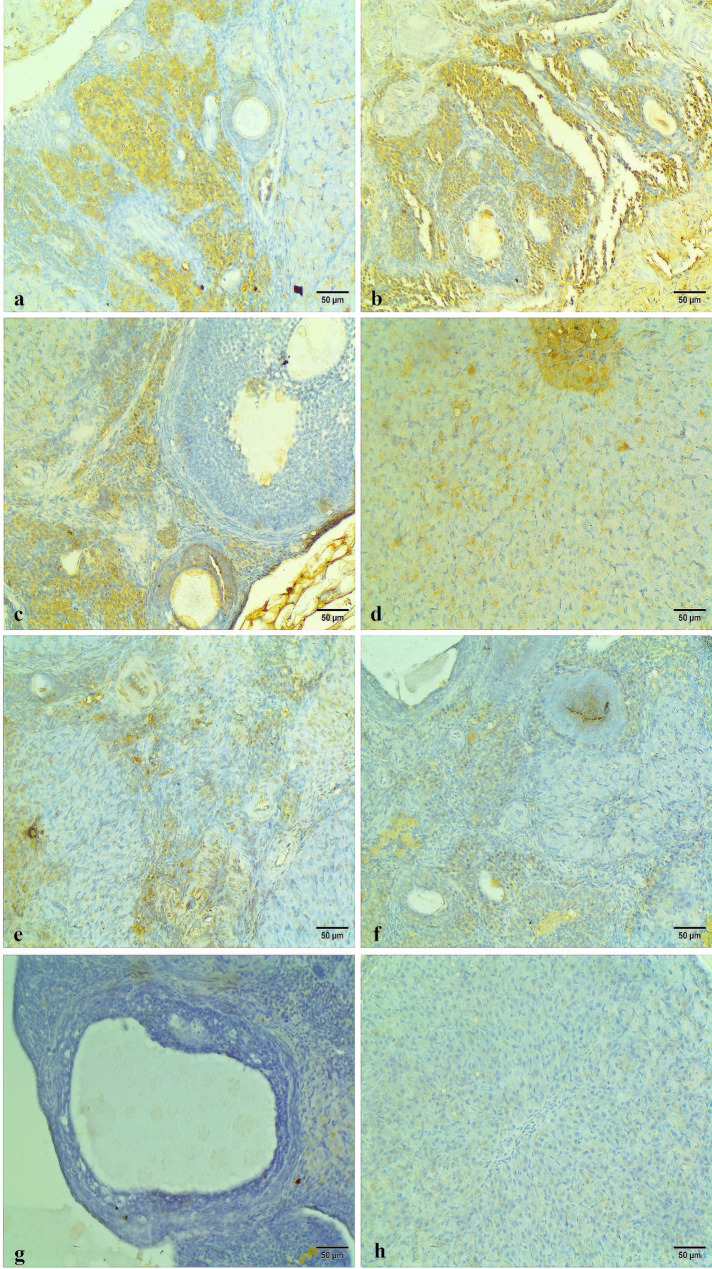
Immunohistochemical expression of p-Akt of ovarian tissues of the Cyclo-treated group showing high p-Akt immunoreactivity within the ovarian cortex (**a**), ovarian medulla (**b**), Graafian follicle (**c**), and corpus luteum (**d**). Cyclo + Carva-treated group showing low p-Akt positivity within the ovarian cortex (**e**), ovarian medulla (**f**), Graafian follicle (**g**), and corpus luteum (**h**). (Immunohistochemical staining Scale bar 50 μm). Carva; carvacrol, Cyclo; cyclophosphamide

**Fig. 14:**
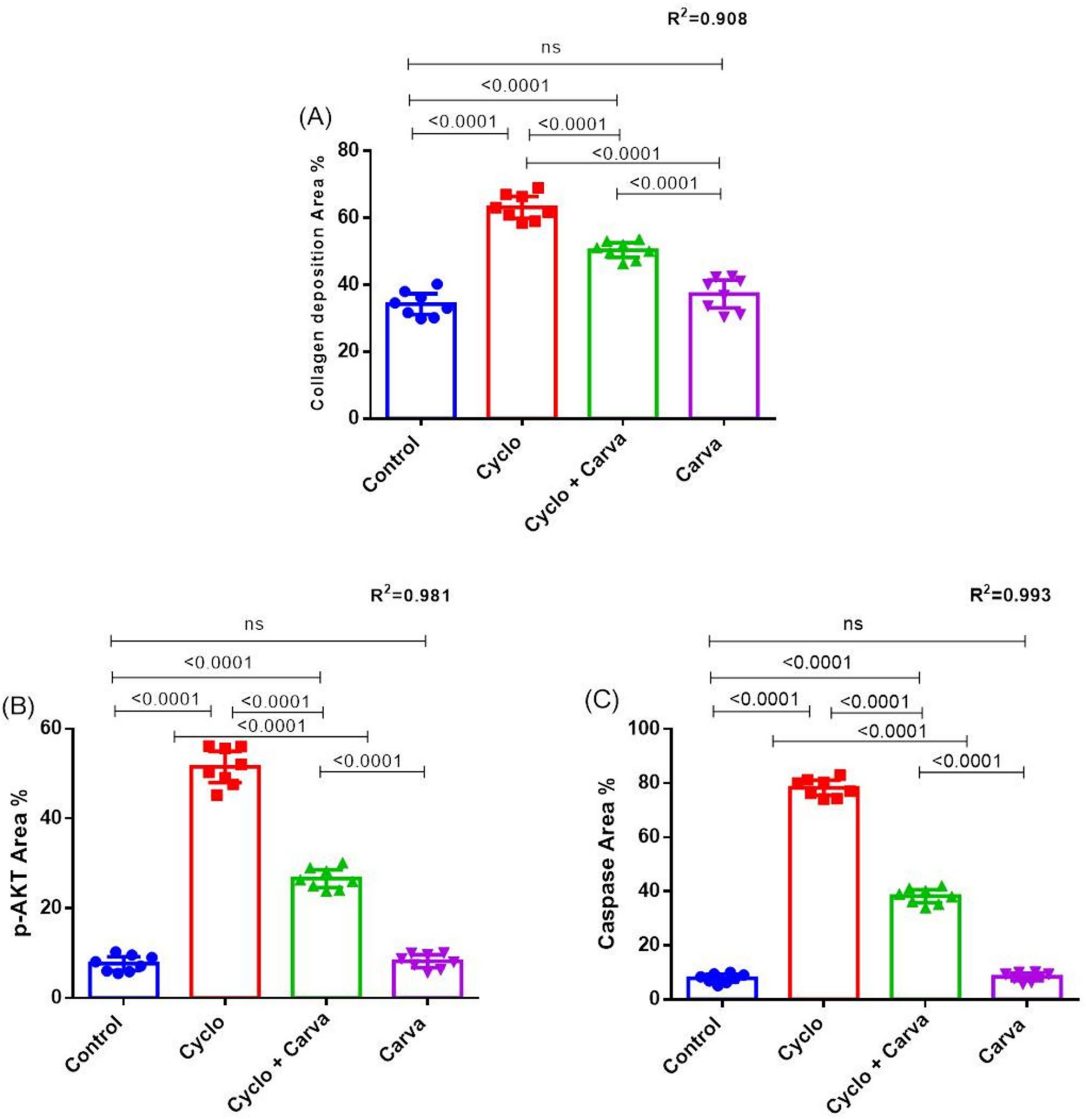
**A** Collagen deposition area % of in the uterus tissues of different groups. Immunostaining expression of and p-AKT (**B**) and caspase 3 (**C**) proteins area % in ovarian tissues of different groups. Values are presented as mean ± CI (*n* = 8 fields). One way ANOVA followed by Tukey’s test for multiple comparisons was applied. Carva; carvacrol, Cyclo; cyclophosphamide

Moreover, the p-AKT immunohistochemical staining appeared as intracytoplasmic brownish colorations within the ovarian cortex, medulla, and different follicles. The Control and Carva-treated groups exhibited low to mild p-AKT immunostaining of the ovarian tissues (Fig. 12). Conversely, high positive p-Akt immunostaining was observed in the ovarian tissue sections of the Cyclo-treated group as shown in Figs. 13a, b, c, and d. Contrary to that, the ovarian tissue of the Cyclo + Carva group showed low to mild p-AKT reactivity when compared to the Cyclo-treated group (Figs. 13e, f, g, and h). Moreover, the area percentage of p-AKT immunostaining in the different groups is represented in Fig. 14B.

## Discussion

Chemotherapy may cause young women and prepubertal adolescents with cancer to experience iatrogenic POF and fertility loss [42]. Individualized strategies, incorporating both established and experimental techniques, should be provided to women diagnosed with cancer to prevent the loss of ovarian function and fertility. These strategies should take into account the patient’s age, marital status, economic status, chemotherapy regimen, type of cancer, staging at diagnosis, and likelihood of treatment delay [43]. Sufficient interdisciplinary oncofertility methods are essential to deliver excellent care to cancer patients. Young cancer patients, especially those in low- and middle-income countries, still have limited access to fertility preservation despite the growing interest and advancements in oncofertility therapies [42]. Thus, searching for alternative effective, safe, and affordable approaches to protect the gonads from the toxic impacts of chemotherapy is highly encouraged. In this manner, our study has been established to elucidate the potential role of Carva in maintaining the rat ovarian structure and function during the administration of chemotherapeutic agent; Cyclo.

As extensively documented in the literature, Cyclo has accountable and serious influences on the female reproductive system [5]. Cyclo is a well-known facilitator of a considerable reduction in the ovarian reserve [44]. Some studies indicated that oxidative stress resulting from Cyclo is a primary cause of ovarian dysfunction through the disturbance of the PI3K/PTEN/AKT signaling pathway [4, 45]. As previously reported, Cyclo could induce POF condition by inducing apoptosis of highly proliferating granulosa cells surrounding ovarian follicles during growth, which resulted in ovarian atrophy, ovarian interstitial fibrosis, and lastly oocyte death [46].

Carvacrol is a type of natural essential oil in several aromatic plants and is well known for its numerous biological activities such as antioxidant, immune-modulatory, hepatoprotective, anti-cancer, diabetes prevention, cardio-protective, and anti-obesity [15, 47]. Multiple piece of evidence highlighted the protective actions of Carva against cellular damage induced by chemotherapy or radiation. For instance, Carva acts as a potential radioprotective agent against X-radiation by neutralizing free radicals and safeguarding cells from radiation-induced injury [48]. Bozkrut et al. [19] and Panahi et al. [23] demonstrated Carva’s protective effects against renal damage provoked by chemotherapeutic agents (Methotrexate or cisplatin, respectively). Similarly, other investigations reported that Carva alleviates kidney, cardiac, and testicular dysfunction associated with Cyclo exposure by reducing oxidative stress [20, 21] and [22]. Furthermore, in a prior study, the protective effects of Carva were reported against Cyclo-induced hepato-renal toxicity in rats [49]. Moreover, previous study revealed the ovario-protective potential of Carva in cisplatin rat model [50]. From this point, the present study was established to speculate on the potential protective impact of Carva against Cyclo-induced rat POF, a model indicative of the effects of chemotherapeutic drugs used in clinical trials [51]. Assessments of serum sex hormones, as well as ovarian oxidative stress-mediated PTEN/PI3K/AKT/FOXO3a/caspase3 signaling pathways, along with histopathological alterations in ovary and uterine tissues, have been investigated to look into the possible mode of action or underlying mechanisms that Carva could affect. In addition, monitoring the rats’ estrous cycle was performed via vaginal cytology.

Oxidative stress is a crucial stimulator of the incidence of POF upon Cyclo treatment. This might be attributed to suppression of antioxidant enzyme expression, boost of free radicals generation with profound lipid peroxidation, and DNA damage in ovarian tissues [7]. All these factors participate in reducing ovarian function, oocyte quality, and ovarian reserve [52]. Thus, reducing oxidative stress leads to dampening follicular atresia and delaying POF. Our findings supported those of Hu et al. [53] and Abdoon et al. [11] and demonstrated that Cyclo promotes ovarian oxidative stress, which is defined by a large drop in the amount of ovarian GSH, and an increase in ovarian MDA level, an indicator of lipid peroxidation. On the other side, the ability of Carva to reverse the condition of ovarian oxidative stress induced by Cyclo was validated in our study, as demonstrated by the significant decline in the MDA amount and partial restoration of GSH activity in the ovarian tissues. Our results are in agreement with those of a previous study that documented the beneficial impacts of Carva on reducing oxidative stress-induced by cisplatin (a well-known chemotherapeutic agent) in rats’ ovarian tissue [50]. The antioxidant properties of Carva were earlier investigated and highly referred to its hydroxyl group giving the ability to scavenge radicals like superoxide radicals, nitric oxide, and hydrogen peroxide [54, 55].

In addition, there was a considerable increase in the concentrations of serum FSH and LH in the Cyclo group, while the contents of serum AMH, estradiol, progesterone, and total estrogen were markedly lower than those in the control group, which was generally consistent with the clinical manifestations of POF occurrence, as reported by former studies [56, 57]. By contrast, Carva significantly ameliorated the toxic influences of Cyclo on these hormones, as evidenced by elevating the serum levels of AMH, estradiol, progesterone, and total estrogen along with partial normalization in the serum levels of FSH and LH in the Cyclo + Carva-treated rats. The results obtained by a recent study are in line with our findings that documented the ability of Carva to preserve the hormonal balance with a successful ovarian reserve in a rat model of polycystic ovarian syndrome [58].

The interplay between FSH and LH has been found to possess a central role in the process of follicular development in the ovary [59]. Though FSH promotes follicular growth and development [60], LH levels rise quickly as follicles expand dominantly, encouraging the formation of more follicles [61]. It is commonly known that AMH is an accurate indicator of ovarian reserve [3]. Hence, the observed rise in serum AMH level of Cyclo + Carva-treated rats supported the results of morphometric analyses, which indicated a rise in ovarian follicle counts with a significant reduction in follicle death, reflecting the efficacy of Carva to preserve the ovarian function. Additionally, the protective action of Carva against Cyclo-caused POF may be attributed to its estrogen-like activity, as earlier reported [62].

Our experimental findings demonstrated that Cyclo induction of POF resulted in a sharp decrease in the number of normal follicles, indicating a possible increase in the frequency of ovarian injury which agrees with former research [63–65]. The PTEN/PI3K/AKT signaling pathway plays a crucial role in proper cell growth, proliferation, and apoptosis for the granulosa layer around the oocyte and it’s considered essential for coordinating oocyte development with granulosa cell proliferation [66]. The balance between positive and negative regulation of the PTEN /PI3K/AKT pathway is important for maintaining the calm state of follicular cell growth [67]. Oocyte-specific PTEN inhibition by phosphorylation to p-PTEN induced activation of PI3K/AKT as PTEN’s action as a negative regulator of PI3K [68]. Specifically, phosphorylation of PTEN at Ser380 (within the C-terminal tail cluster) is generally regarded as a stabilizing but inhibitory modification, reducing its lipid-phosphatase activity and thereby facilitating PI3K/AKT pathway activation [69, 70]. In our study, changes in p-PTEN (Ser380) levels should therefore be interpreted as reflecting altered inhibitory regulation of PTEN rather than straightforward activation. Once PI3K is activated by phosphorylation to p-PI3K, it can phosphorylate and activate a chain of pathways, this means, activating AKT by phosphorylation to p-AKT [71].

Furthermore, FOXO3 is the prime effector of the PTEN/PI3K/AKT pathway in the context of primordial follicle activation. Removal of FOXO3a from the oocytes of primordial follicles results in full activation of primordial follicles leading to infertility [72]. FOXO3a overexpression inhibits follicle activation. When oocyte FOXO3a is overexpressed, it develops infertility due to oocyte growth and follicle retardation [71]. Activation of PTEN/PI3K/AKT signaling inhibits FOXO3a by phosphorylation to p-FOXO3a and induces its transfer from the nucleus to the cytoplasm, inactivating the transcriptional activity of FOXO3a [73], culminating in primordial follicle activation and POF.

In this investigation, our results verified the elevated p-PTEN (Ser380) is consistent with decreased PTEN lipid-phosphatase function and therefore with increased PI3K/AKT pathway activity in the ovarian tissue of the Cyclo group coupled with a subsequent increase in the ovarian p-FOXO3a level, intensifying the incidence of POF. These outcomes are in agreement with former research that deduced the direct impact of Cyclo on the PTEN/PI3K/AKT/FOXO3a pathway [9, 71, 74]. On contrary, Carva treatment suppressed the ovarian PI3K/AKT/FOXO3a pathway through reduction in ovarian p-PTEN (Ser380) expression which would suggest relief of its inhibitory modification. In agreement with former reports, it could be implied that the PTEN/PI3K/AKT/FOXO3a signaling pathway may be involved in the reported therapeutic effects of Carva [25, 75].

Explicitly, in the context of ovarian follicle dynamics, FOXO3a is known to regulate genes involved in apoptosis and follicular atresia. AKT-mediated phosphorylation of FOXO3a reduces its nuclear activity, thereby favoring follicle survival [76]. In our results, Cyclo treatment increased p-AKT and p-FOXO3a, while Carva reversed both, suggesting that Carva attenuates AKT signaling and restores FOXO3a activity, which may contribute to the observed reduction in apoptosis and preservation of follicular integrity.

Furthermore, high levels of oxidative stress can stimulate PI3K/AKT signaling [77, 78], and influence several cellular stress responses, including apoptosis [79]. The pro-apoptotic signals are increased by the excess generation of free radicals that bind to DNA [80]. This signal is characterized by the entry of mitochondrial cytochrome c into the cytoplasm, which triggers caspase-3 and encourages cell death [81]. In this research, Cyclo was found to induce apoptosis in the ovary, as demonstrated by strong caspase 3 expressions in the ovarian cortex, corpus luteum, and medulla, as well as in the secondary and Graafian follicles. This apoptotic action of Cyclo could be attributed to the profound oxidative stress that is linked to the hyper-activation of ovarian p-AKT expression. Moreover, this result explains the observed follicular atresia in the Cyclo group. These outcomes are parallel to those of Davis and Heindel [82], Desmeules and Devine [83] and Liang et al. [84].

Contrarily, Carva has the potential to prevent apoptosis through combating oxidative insult, as evidenced by the reduction of caspase 3 and p-AKT expressions in the different ovarian components, supporting its anti-apoptotic/antioxidant action. This result comes in line with other research [21, 85].

Parallel to prior scholars, rats intoxicated with Cyclo revealed severe damage in ovarian system architecture, as demonstrated by the histopathological results [86]. Regarding the ovary, vascular congestion and hemorrhage in the ovarian stroma, as well as hemorrhage around the corpora lutea with subsequent ovarian atrophy, in addition to follicular atresia and follicle depletion were observed in the Cyclo-challenged group. Conspicuously, a significant reduction in rats’ body weight and reproductive tract weight has been recorded in the Cyclo-intoxicated group, which is in line with a previous report [87]. Moreover, the uterine tissue sections of rats intoxicated with Cyclo showed vascular congestion, epithelial degeneration, and vacuolization in epithelial cells, inactive low cuboidal to the flattened epithelial lining, alongside diminished inactive endometrial glands, fibrotic stroma, and glandular proliferation, and uterine diameter. The condensation of collagen fibers within the uterine tissue parenchyma following Cyclo administration was also verified from Masson’s trichrome staining findings which agrees with a former research [88]. The development of uterus fibrosis in the Cyclo group could be linked to the observed low estrogen level. Where there are strong hormonal interdependences between the ovaries and uterus. One important function of estrogen synthesized by the ovaries is to preserve normal reproductive organ tissues like those in the uterus [89]. In such cases, when there is primary ovarian insufficiency, low estrogen levels may lead to some changes in the uterus including fibrotic lesions [90] as detected in our study. Accordingly, a marked disturbance in the estrous cycle was detected in rats intoxicated with Cyclo, as shown by the extension of the estrous cycle by up to 8–10 days, than the control group. The observed hormonal imbalance upon Cyclo toxicity could explain the disturbance of the rat’s estrous cycle. Conversely, oral administration of Carva to Cyclo-intoxicated animals significantly improved all these negative impacts of Cyclo on ovarian tissue structure, fibrosis of uterine parenchyma, as well as rat’s body weight and reproductive tract weight. Furthermore, the preservation of the regular estrous cycle of rats treated with Cyclo + Carva supported the beneficial effects of Carva in maintaining the female reproductive function, and hormonal balance during chemotherapy.

The observed increase in uterine weight and endometrial granularity in the Carva -treated control group indicates a potential uterotrophic effect [91]. This suggests that Carva may possess weak estrogenic or estrogen-mimetic properties, a phenomenon supported by the findings of a previous research [92] demonstrating that Carva administration significantly increased uterine estrogen receptor-alpha (ER-α) expression in rats. This proliferative effect critically confounds the interpretation of “protection” against Cyclo-induced damage. The improved endometrial morphology in the Cyclo + Carva cohort may not be due solely to direct cytoprotection but could represent a pharmacological override, whereby the compound’s stimulatory action masks the underlying cytotoxic injury by promoting tissue growth. Consequently, the net improvement in histological endpoints is likely the result of competing apoptotic (Cyclo) and proliferative (Carva) signals, rather than a pure attenuation of toxicity. Also, our histological analysis shows that while the carvacrol-only group has thickened epithelium, the Cyclo + Carva group shows signs of reduced inflammation and apoptosis compared to Cyclo -only, which is not solely explained by estrogenicity. Hence, this study directly demonstrates that carvacrol administration increases uterine weight and improves endometrial morphology in a rat model, specifically noting its proliferative and restorative effects. The authors attribute this to its antioxidant and anti-apoptotic properties but also explicitly discuss its potential phytoestrogen-like activity as a contributing mechanism. In this context, future studies employing ovariectomized models and/or co-treatment with selective estrogen receptor antagonists will be essential to distinguish whether Carva’s apparent benefit arises from true cytoprotection or from estrogenic stimulation that masks underlying toxicity.

## Conclusion

Our research’s findings could deduce the efficacy of Carva in improving ovarian damage and uterine fibrosis during chemotherapy treatment. Oral administration of Carva (15 mg/kg) preserved the rat ovarian function by shielding the ovaries from oxidative stress-induced ovarian apoptosis via modulating the p-PTEN/p-PI3K/p-AKT signaling pathway, suppressing FOXO3a nuclear shuttling and subsequently impeding POF and hormonal imbalance in a Cyclo rat model. However, more comprehensive investigations could be established to investigate the efficacy of Carva in preservation and restoring female fertility during and after chemotherapy. Furthermore, extensive future studies are highly recommended to explore other possible mechanisms by which Carva could maintain ovarian function using other experimental approaches with chemotherapy. Lastly, future research is needed to study the interfering possibility of Carva with the effectiveness of chemotherapy in experimental cancer models.

## Limitations of the study

A limitation of the present study is the use of different analytical platforms to quantify phosphorylated signaling proteins (western blot for p-PI3K/p-PTEN and ELISA for p-AKT/p-FOXO3a). This approach was chosen to balance assay sensitivity, quantitative precision, and the limited sample volumes available. Although both ELISA and western blot are well-established for phosphoprotein measurement, platform differences complicate absolute, cross-platform comparisons of phosphorylation magnitude. We mitigated this by (i) using validated ELISA kits with documented methods, (ii) applying consistent normalization procedures (β-actin for blots; total protein for ELISA), and (iii) corroborating phosphorylation changes with complementary outcome measures (histology and biochemical assays). However, future studies are required to perform parallel western blot analyses for p-AKT and p-FOXO3a. Furthermore, a limitation of this study is that phosphorylation changes were normalized only to β-actin and not to the corresponding total protein levels (e.g., total PI3K, PTEN). As such, we cannot definitively distinguish between changes in phosphorylation stoichiometry and alterations in protein abundance. While β-actin normalization provides a reliable control for loading, conducting future studies to incorporate total-protein blots or quantitative ELISAs to strengthen mechanistic interpretation are required. An important translational consideration is the potential pharmacokinetic interaction between Carva and Cyclo. Carva and related essential-oil components have been reported to suppress hepatic cytochrome P450 expression and activity in vivo. Because Cyclo requires bioactivation primarily by CYP2B6, with contributions from CYP2C19 and CYP3A4, any CYP inhibition could theoretically attenuate its antitumor efficacy, even while reducing host toxicity. Although our study focused on ovarian protection, further work in tumor-bearing models will be essential to determine whether Carva co-treatment compromises or alters the antineoplastic efficacy of Cyclo.

## Supplementary Information

Supplementary Material 1.

Supplementary Material 2.

Supplementary Material 3.

Supplementary Material 4.

## Data Availability

All data generated or analyzed during this study are included in this published article.

## Abbreviations

AKT: Protein kinase B
AMH: Anti-Mullerian hormone
ANOVA: Analysis of Variance
bw: body weight
Carva: Carvacrol
CI: Confidence intervals
Cyclo: Cyclophosphamide
EDTA: Ethylenediaminetetraacetic acid
FOXO 3a: Forkhead box protein O3a
FSH: Follicle-stimulating hormone
H and E: Hematoxylin and Eosin (H&E)
LH: Luteinizing hormone
PI3K: Phosphoinositide 3-kinases
POF: Premature ovarian failure
PTEN: Phosphatase and tensin homolog protein
