# Supplementary material for: Carvacrol ameliorates cyclophosphamide-induced rat premature ovarian failure and uterine fibrosis via regulating PI3K/AKT/FOXO3a signaling pathway
Source: J Ovarian Res. 2025 Dec 9;18:291. doi: 10.1186/s13048-025-01880-3 (PMC12687504; doi:10.1186/s13048-025-01880-3)
Supplement: Supplementary file 1 — Supplementary Material 1. [file 13048_2025_1880_MOESM1_ESM.docx]

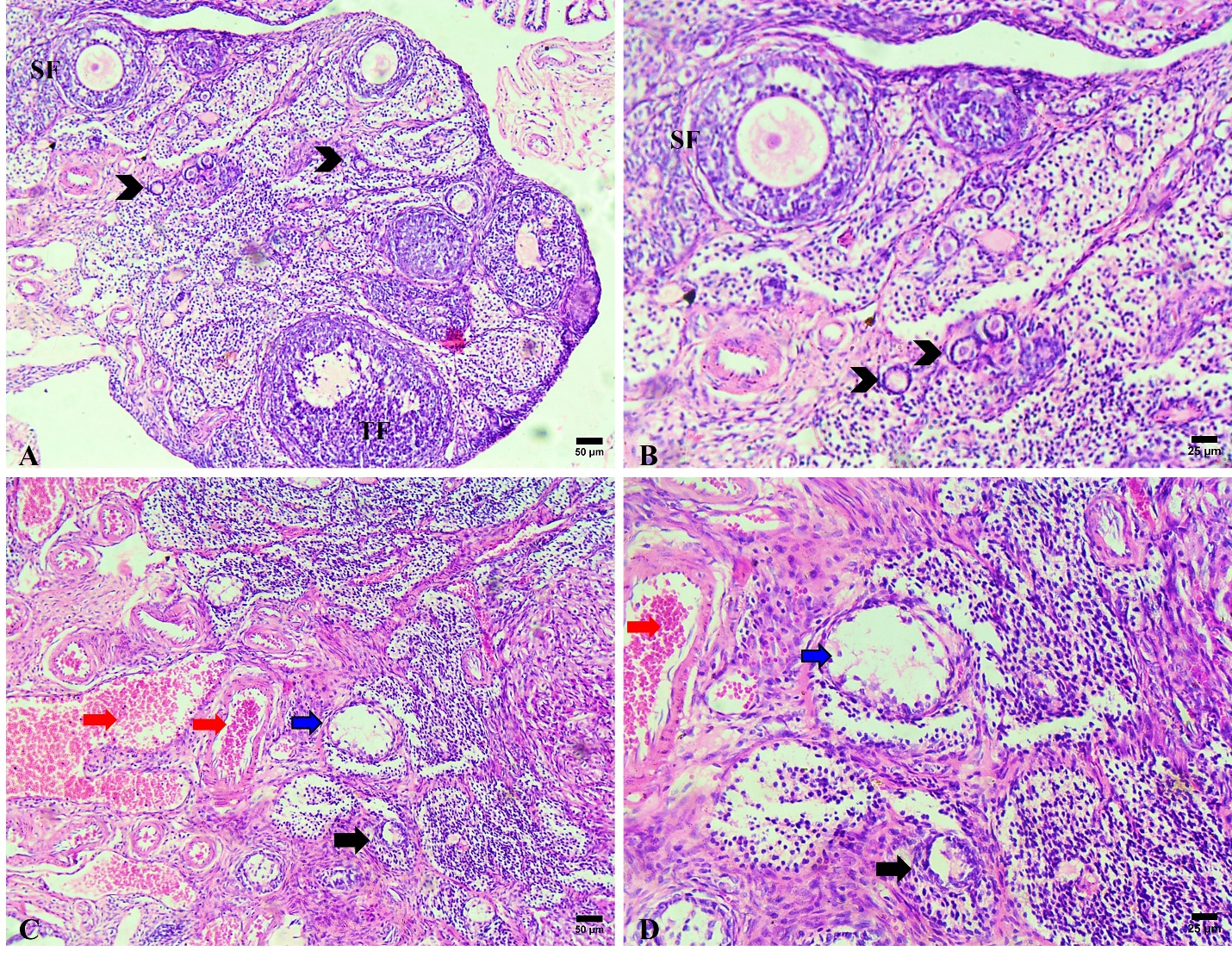


**Fig. 1S:** Photomicrograph of ovarian tissue of Carva treated- rats (A, B) showing primary follicle (Arrow head), secondary follicle (SF), and tertiary follicle (TF). Cyclo treated- rats (C, D) showing moderate ovarian venous congestion and hemorrhages (Red arrows) with degenerated primordial follicles (black arrow) and secondary follicle (Blue arrow) as well as apoptotic bodies (red star). (H&E A, C 50 µm magnification, B, D 25 µm magnification).
