# Supplementary figures and images for: Carvacrol ameliorates cyclophosphamide-induced rat premature ovarian failure and uterine fibrosis via regulating PI3K/AKT/FOXO3a signaling pathway
Source: J Ovarian Res. 2025 Dec 9;18:291. doi: 10.1186/s13048-025-01880-3 (PMC12687504; doi:10.1186/s13048-025-01880-3)

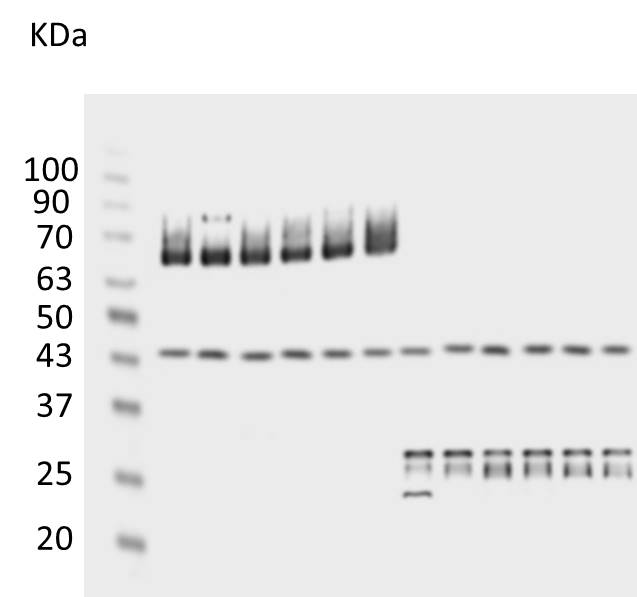

Supplement: Supplementary file 2 — Supplementary Material 2. [file 13048_2025_1880_MOESM2_ESM.jpg]

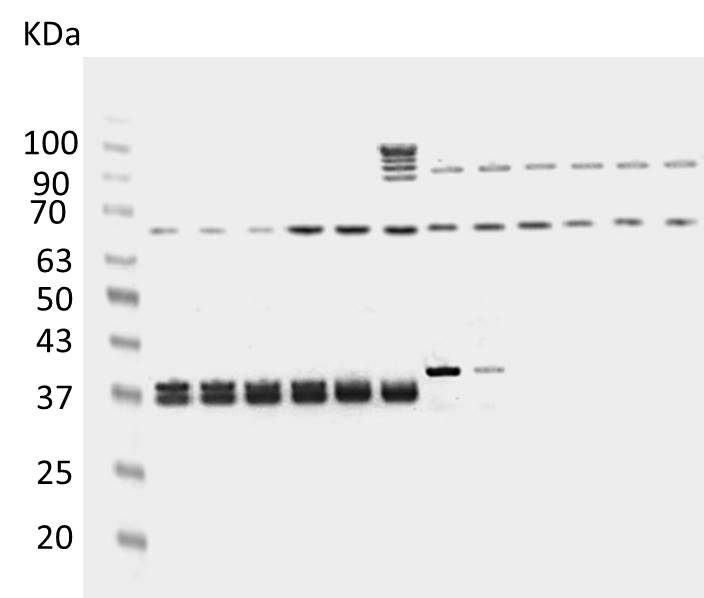

Supplement: Supplementary file 3 — Supplementary Material 3. [file 13048_2025_1880_MOESM3_ESM.jpg]

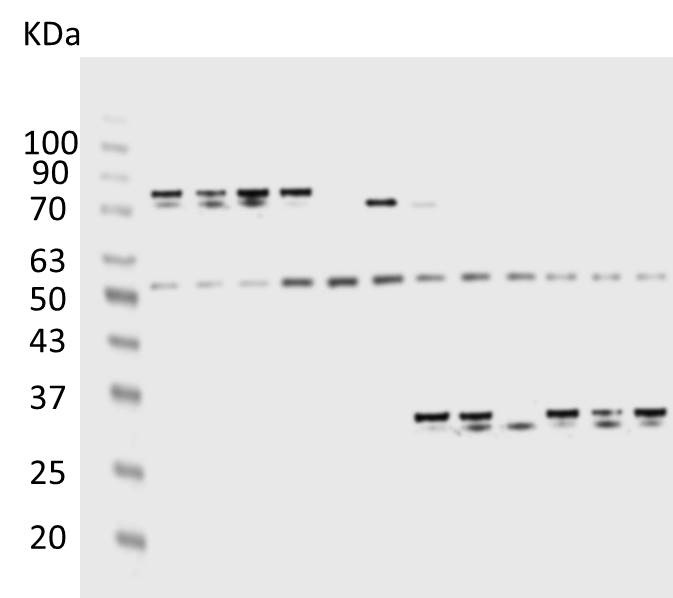

Supplement: Supplementary file 4 — Supplementary Material 4. [file 13048_2025_1880_MOESM4_ESM.jpg]
